# Adverse health outcomes associated with drinking highly saline water: a systematic review

**DOI:** 10.1007/s10654-025-01307-9

**Published:** 2025-09-29

**Authors:** Eurydice Costopoulos, Airi Imamura, Nushrat Khan, Adrian Butler, Christopher Millett, Mohammad A. Hoque, Paolo Vineis, Kristine Belesova, Aneire Khan

**Affiliations:** 1 Department of Epidemiology and Biostatistics, School of Public Health, Imperial College London, 90 Wood Lane, London, W12 0BZ UK; 2 School of Medicine, University College Dublin UCD Health Sciences Centre, Belfield, Dublin 4, D04 V1W8 Ireland; 3 Public Health Policy Evaluation Unit, School of Public Health, Imperial College London, 90 Wood Lane, London, W12 0BZ UK; 4 Department of Civil and Environmental Engineering, Imperial College London, Exhibition Road, London, SW7 2BU UK; 5 Public Health Research Centre & Comprehensive Health Research Center (CHRC) National School of Public Health NOVA University of Lisbon, Lisbon, Portugal; 6 School of the Environment and Life Sciences, University of Portsmouth, Portsmouth, PO1 2UP UK

**Keywords:** Climate change, Salinity, Drinking water, Cardiovascular health, Renal health, Maternal and infant health.

## Abstract

In climate change-affected coastal areas, sea level rise, storm surges, droughts and altered rainfalls are significantly increasing salinity levels in drinking water sources. This is a major public health problem that affects many millions of people. We systematically reviewed and assessed the strength and quality of the evidence on the relationship between drinking water with high sodium levels (> 200 mgNa/l) and adverse cardiovascular, renal, and pregnancy-related health outcomes, following the PRISMA guidelines, the ROBINS-E Cochrane tool and the Navigation Guide. From five bibliographic databases, we identified 22 relevant studies, some of which assessed more than one health domain. The evidence was of moderate quality and strength. 14 analyses from eight studies at low risk of bias and four studies at moderate risk of bias, linked drinking high-salinity water to adverse health outcomes including hypertension and cardiovascular disease, impaired renal function, gestational hypertension and preeclampsia, and higher infant mortality. Eight studies were inconclusive. Three analyses, of which two at low risk of bias, associated drinking high-salinity water to improved health outcomes. Overall, our findings suggest that salinisation of drinking water sources is likely to increase adverse cardiovascular, renal, and pregnancy-related health outcomes. This conclusion highlights the importance of effective and timely adaptation at scale, and calls for a revision of the WHO guidelines for the intake of salt from water. The latest WHO guidelines (2022) do not set any health-based standard for sodium levels in drinking water, a problem that affects millions of people and will worsen with climate change.

**Supplementary Information:**

The online version contains supplementary material available at 10.1007/s10654-025-01307-9.

## Background

40% of the world’s population lives within 100 km of a coastline [1]. Coastal population density is steadily rising, which increases the pressure on coastal freshwater resources [1]. Additionally, altered rainfall patterns and river flows directly impact the replenishment of freshwater, while rising sea levels, cyclones, and storm surges lead to saltwater infiltration into previously non-saline reserves [2–4]. Fluoride contamination of the aquifers also causes high sodium levels in water [5]. These trends are driving an expansion in the geographical range of areas experiencing drinking water salinisation, as well as increasing intensity of salinisation in areas already affected [2–4]. The impacts disproportionately affect the poorest and most vulnerable communities in many low- and middle-income countries [2, 3].

Excessive dietary sodium intake has been linked to multiple adverse health outcomes including hypertension, cardiovascular disease and kidney disease [6]. The World Health Organization (WHO) recommends consuming no more than 2,000 milligrams of sodium (mgNa) per day – yet the mean global (mainly dietary) intake for adults is 4,310 mgNa [6]. In 2021, diets high in sodium accounted for 1.86 million deaths and 41.3 million disability adjusted life years (DALYs) worldwide, primarily due to cardiovascular diseases [7]. To date, most of the existing evidence pertains to dietary sodium consumption. Epidemiological research on the health impacts of excessive sodium intake from drinking water is nascent, yet evidence syntheses in this area are limited. As a result, the latest WHO guidelines (2022) do not set any health-based standard for sodium levels in drinking water, merely noting that concentrations above 200 mgNa/l would result in an unpleasant taste [8]. The absence of guidelines hinders the development and implementation of policies and actions to mitigate excessive drinking water salinity.

In high-income countries, sodium levels in drinking water are typically considered negligible due to widespread access to high-quality water. However, this may not be the case in many low- and middle-income countries that lack the infrastructure and resources to effectively treat and distribute water [2]. For example, in Bangladesh, approximately 20 million people live in salinity-affected areas [9, 10], where sodium levels in drinking water can exceed 600 mgNa/l [11–14]. Similarly, in Kenya, Lake Turkana is periodically used as a drinking source, despite salinity levels reaching 920 mgNa/l [15]. However, given the presence of several ions in water, high levels of sodium cannot always be interpreted separately from the other minerals present. While some of these ions, such as sodium, could be detrimental to human health, others, such as calcium, magnesium and potassium, may have cardioprotective effects [16–18]. In highly-mineral water, therefore, it is difficult to evaluate sodium’s effect alone.

An emerging body of literature suggests a potential association between the consumption of highly saline water and adverse health outcomes such as hypertension, miscarriage, skin diseases, acute respiratory diseases and diarrhoeal diseases [2]. A systematic review and meta-analysis published eight years ago found that drinking water with > 150 mgNa/l was associated with elevated blood pressure, particularly diastolic blood pressure, in four out of seven studies analysed [19]. The review synthesised evidence on only one health outcome – blood pressure, and the evidence was only briefly assessed for quality. A recent scoping review looked at the relationship between salts in drinking water (including sodium, potassium, calcium, magnesium, electrical conductivity and total dissolved solids) and blood pressure or hypertension [20]. Of the 29 articles included, half demonstrated a positive, statistically significant, relationship between salts in water and increased blood pressure or risk of hypertension. A quarter demonstrated a non-significant negative association, and another quarter demonstrated a significant negative association, which was mainly attributed to the salubrious effects of potassium, calcium and magnesium, rather than sodium. New studies on the association of drinking water salinity with other health outcomes besides cardiovascular ones, e.g., renal function, maternal health and child health, have become available since, but have not been systematically synthesised yet [15, 21–29].

**Fig. 1:**
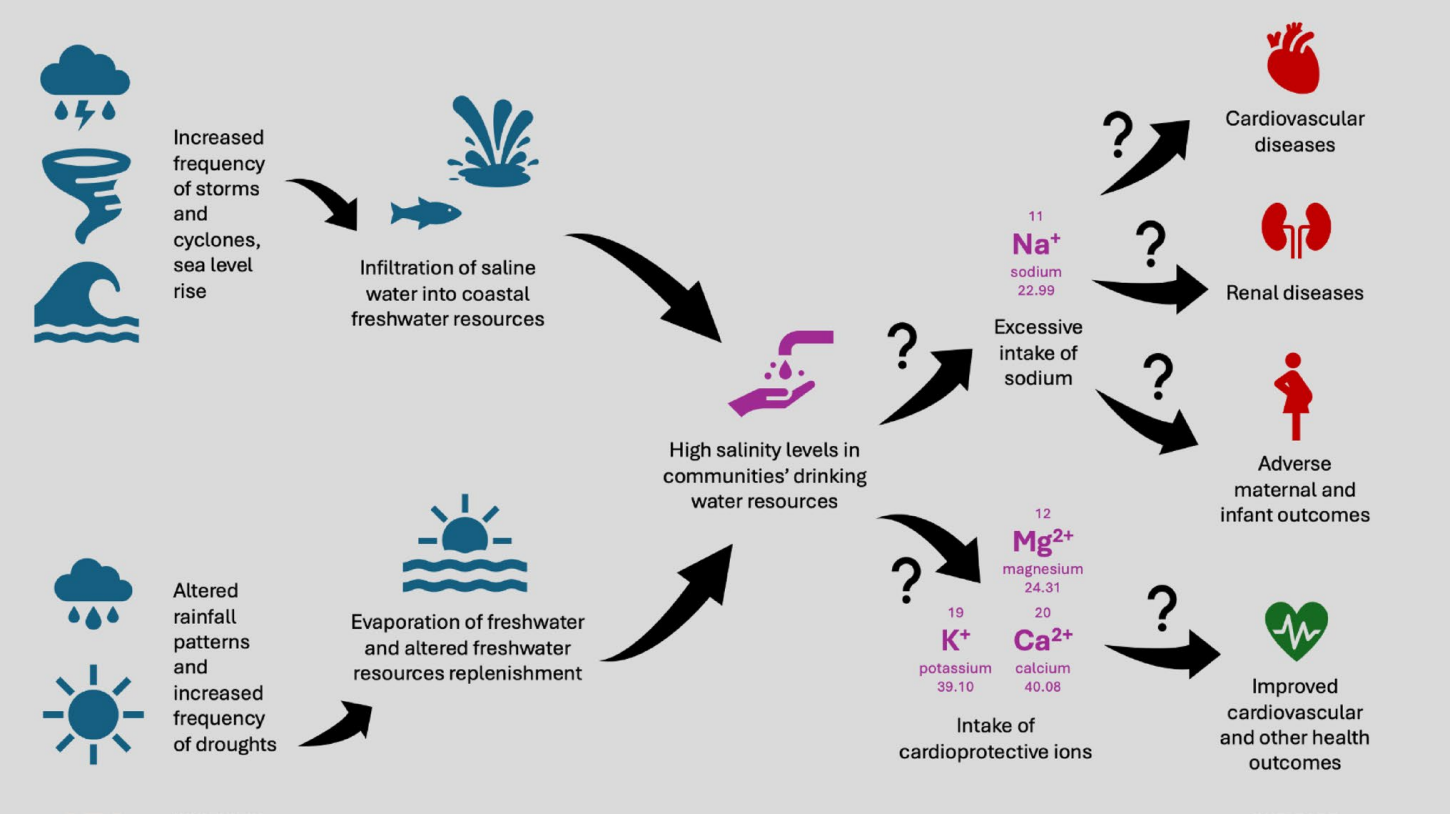
Potential, but unquantified, health consequences of chronic drinking of high-salinity water due to climate change

High sodium levels in drinking water sources are becoming an increasingly important issue in many low- and middle-income countries, with climate change exacerbating the problem and putting millions more at risk [2]. Figure 1 illustrates the potential health consequences of chronic drinking of high-salinity water due to climate change. Rising sea levels are on a trajectory of increasing salinisation of drinking water through the end of the century, even with successful mitigation efforts [2]. As it is often the case, the poorest and most vulnerable communities are disproportionately affected. This systematic review aims to synthesise and provide a comprehensive assessment of the quality and strength of evidence on the association between drinking high-salinity water and three health outcome categories: cardiovascular health, renal function, and pregnancy-related outcomes. We chose these three domains - rather than all the health outcomes considered in published papers - because the literature on the other health outcomes was sparse and usually limited to small numbers of observations. Our assessment identifies evidence gaps, methodological strengths and limitations of existing studies, and suggestions for further strengthening of the evidence base to facilitate the development of a health guideline on drinking water sodium content.

## Methods

### Research question

What is the strength of the evidence on the association between drinking high-salinity water and adverse health outcomes (specifically cardiovascular health, renal function and pregnancy-related outcomes)?

### Definition of ‘high-salinity drinking water’

Salinity is a measure of the concentration of total dissolved salts in water [30, 31], usually given in parts per thousand or mg/l. It is directly related to sodium concentration (in mgNa/l), sodium being one of the major ions in water [30, 31]. In coastal waters dominated by sodium chloride, as is the case of many studies examined here, sodium constitutes around 40% of the total mass of dissolved ions [32]. Wherever necessary, therefore, we multiplied salinity measurements by 0.4 to obtain the equivalent sodium concentration. Electrical conductivity (in milli-Siemens or micro-Siemens per centimetre, mS/cm or µS/cm) is closely correlated to salinity, as the dissolved ions are the conductors of electrical current [31]. Finally, total dissolved solids can be calculated by adding the concentrations of all individual ions, or by multiplying the electrical conductivity by a factor dependent on local water’s temperature and chemistry [32]. Beyond serving as surrogates for sodium concentration, electrical conductivity and total dissolved solids can be used as estimates of the mineral complexity of water, as they reflect the presence of other, potentially beneficial, salts such as calcium, magnesium and potassium. Yet, as our study focuses on the effects of sodium, these parameters were mainly used as proxies of the sodium content of water. Although chloride concentration is closely associated with sodium levels, the relationship between the two is not always linear, and can vary with time, location and water source [33]. To avoid any approximations and to focus specifically on sodium, measurements of chloride alone were therefore not accepted. As there is no universal cut-off value to define excessive salinity in drinking water, the limits given in Supplementary Table 4 were used (sodium concentration of 200 mgNa/l, for instance), based on the thresholds used by most studies in the field, and on the official drinking water guidelines of the WHO [8], Bangladesh [34, 35], Jordan [36] and the United States [31, 37].

### Search strategy design

We followed the latest PRISMA guidelines and checklist in the design and implementation of the study [38]. The databases searched were Embase, Scopus, Web of Science, Medline and Global Health. Grey literature was not included due to quality concerns and lack of peer-review. Only papers available in English were considered.

The search strategy was elaborated with the help of a specialised librarian. The databases were searched between the 23rd and 26th of February 2024 for studies published after 2000. The search was later extended to include papers published before 2000, with the databases being searched on the 2nd of May 2025. We created five search concepts using both keywords and Medical Subject Headings, and combining them within each concept with ‘OR’. The concepts were: (1) ‘drinking water’; (2) ‘salinity’ and ‘sodium’; (3) ‘pregnancy outcomes’; (4) ‘hypertension’ and ‘cardiovascular diseases’; and (5) ‘renal hypertension’ and ‘kidney disease’. A comprehensive list of alternative terms was included in each. Concepts (3), (4) and (5) were joined together using ‘OR’. The resulting group was linked with ‘AND’ to concepts (1) and (2). A filter was used to exclude animal studies. Supplementary Table 5 contains the full Medline search strategy. This template was adapted to fit the other databases’ search formats.

Supplementary Table 6 displays the full inclusion and exclusion criteria. We only included studies on human populations who chronically consume high-salinity water, as per the definition we established. We excluded papers that did not provide a comparator. The health outcomes assessed had to be either pregnancy-related (maternal or infant), cardiovascular-related, or kidney-related. We excluded case reports, experimental designs (including short-term randomised controlled trials), reviews, qualitative studies, commentaries and abstract reports. We excluded two papers [39, 40], that performed the same analyses on the same datasets as other included papers [12, 23]. We kept the most recent or most complete analysis for each.

This systematic review was registered on PROSPERO; the protocol can be accessed with the number CRD42024512910 [41]. Ethical approval was not necessary.

### Screening process, data extraction and quality appraisal

The search results were imported in Covidence and duplicates were removed [42]. All the titles and abstracts were first screened by two of the authors (EC, AI). A random sample, corresponding to at least 15% of all search results, was triple-screened by more senior researchers (AK, KB, NK). The selected papers then underwent a full-text screening by the two same authors, with senior researchers again randomly triple-screening 15% of the papers that had reached this stage. All reviewers were blind to the decisions taken by their colleagues. When a conflict arose about the inclusion or exclusion of a paper, an additional researcher was asked to make the final decision. When a study could not be retrieved, every possible effort was made to access it, including contacting the Imperial College Library, requesting it on the publication website, or directly emailing the authors. When these steps were unsuccessful, the study was excluded under the heading ‘studies not retrieved’.

Data items extracted included title, author(s), date, study design, country of the study, population(s), exposure(s), comparator(s) and outcome(s). The data was inputted into an Excel file. The risk of bias assessment was performed by the main author (EC), based on the latest version of the ROBINS-E Cochrane tool [43]. This tool, used for non-randomised trials of exposure, accounts for the risk of confounding; co-interventions or post-exposure interventions; the suitability and quality of the exposure measurement and classification; the suitability and quality of the outcome measurement and classification; the risk of bias from the participants selection process, from the randomisation process, from any data missing, and from reporting the results.

The studies’ findings we selected for analysis included: ORs or relative risk ratios of higher blood pressure, hypertension, (pre)eclampsia, prevalence of diseases, hospital visits and mortality, as well as β-coefficients or differences in means or medians of blood pressure values and prevalence of hypertension. We categorised the studies by the health outcomes they examined. The main numerical findings of the studies were inputted in a table with, wherever available, the 95% confidence intervals, the standard deviations and the p-values. We conducted a narrative description and synthesis of the results. Due to the disparity in the methods of exposure measurement, and the wide range of outcomes considered, direct numerical comparison was not possible. Therefore, although considered, a meta-analysis was not performed.

## Results

### Study characteristics

Results of our study selection process are available in the PRISMA [38] flow-chart in Supplementary Fig. 1. From the initial 2,254 search results, 22 papers were included in the final analysis, consisting of 863,470 individual-level data.

Study characteristics are presented in Supplementary Table 1. Over half (*n* = 12) the reviewed studies were set in Bangladesh [9, 11–13, 21–27, 44]. A quarter (*n* = 6) were set in the United States [29, 45–49]. The remaining studies were conducted in Kenya [15], Vietnam [28], Israel [50] and Australia [51]. Population settings differed widely: 13 studies used data from adults in the community [9, 11–13, 15, 21–26, 47, 49]; seven studies examined child data [27, 44–46, 48, 50, 51]; three studies focused on hospital populations [9, 28, 29]. The study designs were heterogeneous, and included descriptive [9, 27, 44], case-control [11, 13, 22, 23, 28, 29], cross-sectional [12, 15, 21, 24, 25, 45–51], cohort [26] and ecological [49] studies.

Salinity exposure was determined based on the sodium concentration in drinking water in most studies (*n* = 19) [9, 11–13, 15, 21–24, 26, 27, 29, 45–51]. Levels of sodium exposure ranged from > 172 mgNa/l [24] to > 2,000 mgNa/l [26] (for definitions of salinity and sodium exposure see above). The remaining studies used various proxies (of varying reliability) to estimate the extent of exposure, including electrical conductivity [25, 44], urinary sodium excretion [25], and province-level data on salinity [28]. Sources of high-salinity drinking water included groundwater [9, 22, 29], pond water [11, 13, 21, 22], and municipal water [29, 45–49, 51]. Water sources used as comparators to sources of higher salinity included rain and filtered water [9, 11, 13, 22], surface water [29], and municipal water [29, 45–49, 51]. The comparators’ salinity ranged from negligible levels [9] to as high as 1,000 mg/l [26], equivalent to 400 mgNa/l. Due to heterogeneity in the categories of sodium concentration between different studies, we adopted the terminology denoting salinity levels (i.e. “high-sodium”, “slightly saline”, etc.) that was originally used by the study authors, alongside quoting the quantitative sodium ranges they referred to.

A total of 16 studies [12, 15, 21–26, 28, 45–51] assessed cardiovascular-related outcomes (cardiovascular disease [24], and blood pressure and hypertension [12, 15, 21–23, 25, 26, 28, 45–51]) in the general (non-pregnant) population. Of these, three studies [15, 21, 22] additionally examined renal function using hyper diluted urine, 24-hour total urinary protein excretion, and self-reported kidney disease as the outcome measures. Six studies [9, 11–13, 27, 29, 44] assessed pregnancy-related health outcome measures: pregnant women’s blood pressure, gestational hypertension, and preeclampsia rates [9, 11, 13, 29]; and child mortality risk in relation to mothers’ consumption of high-salinity water [27, 44].

### Synthesis of the findings

**Fig. 2:**
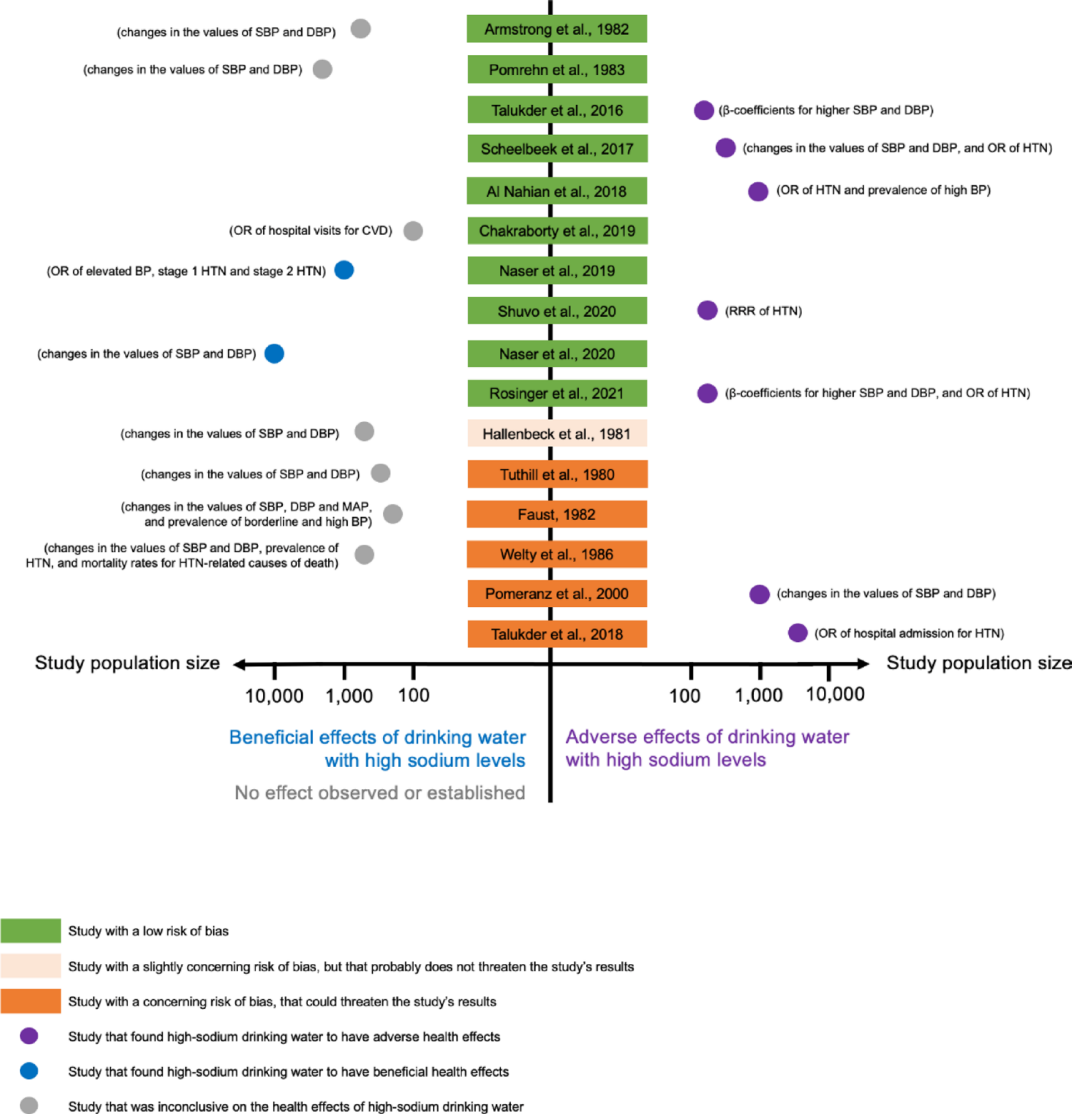
Overall effect on cardiovascular health outcomes found by each study, weighted by the study’s risk of bias score and population size

**Fig. 3:**
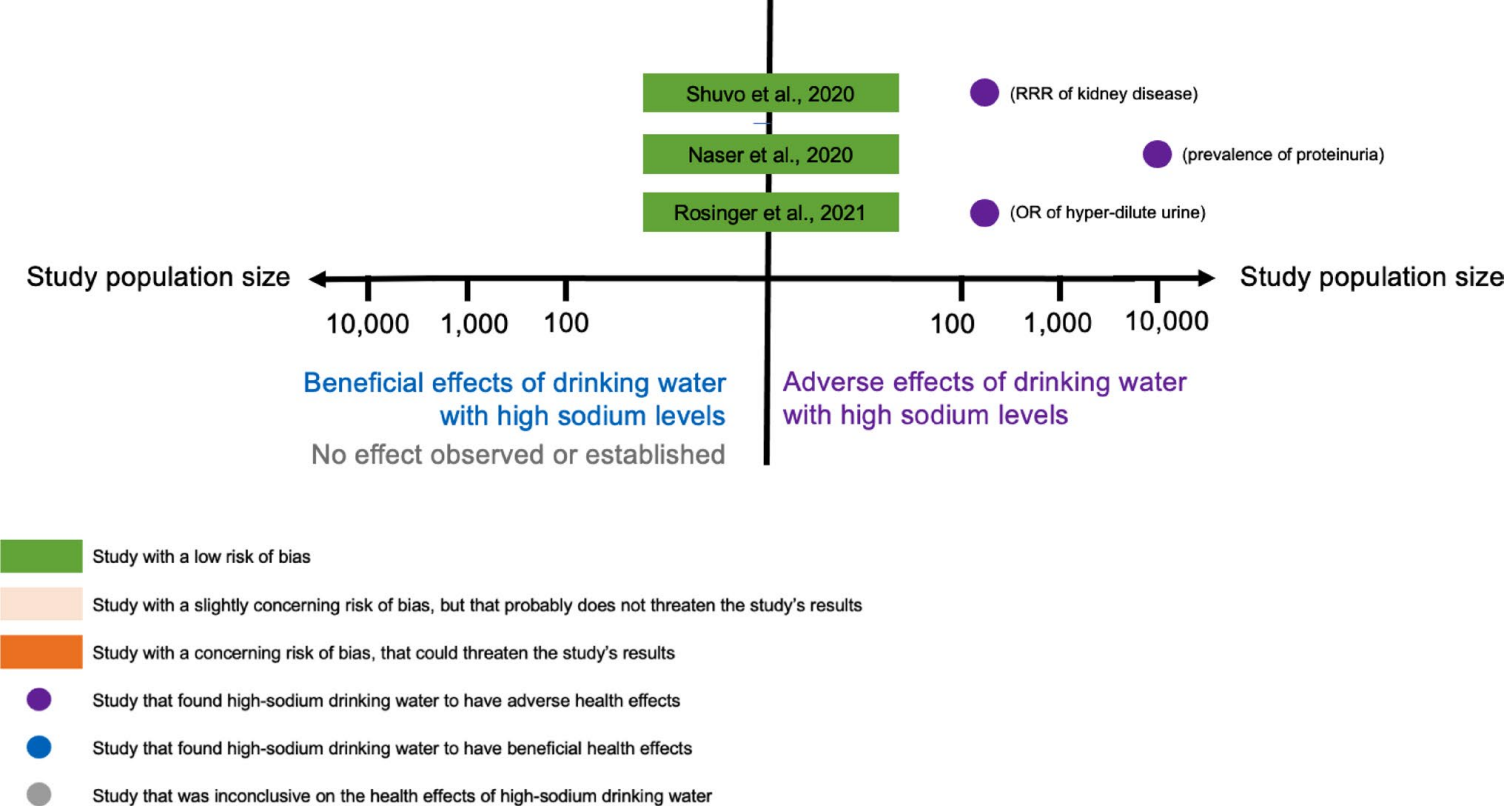
Overall effect on kidney-related health outcomes found by each study, weighted by the study’s risk-of-bias score and population size

**Fig. 4:**
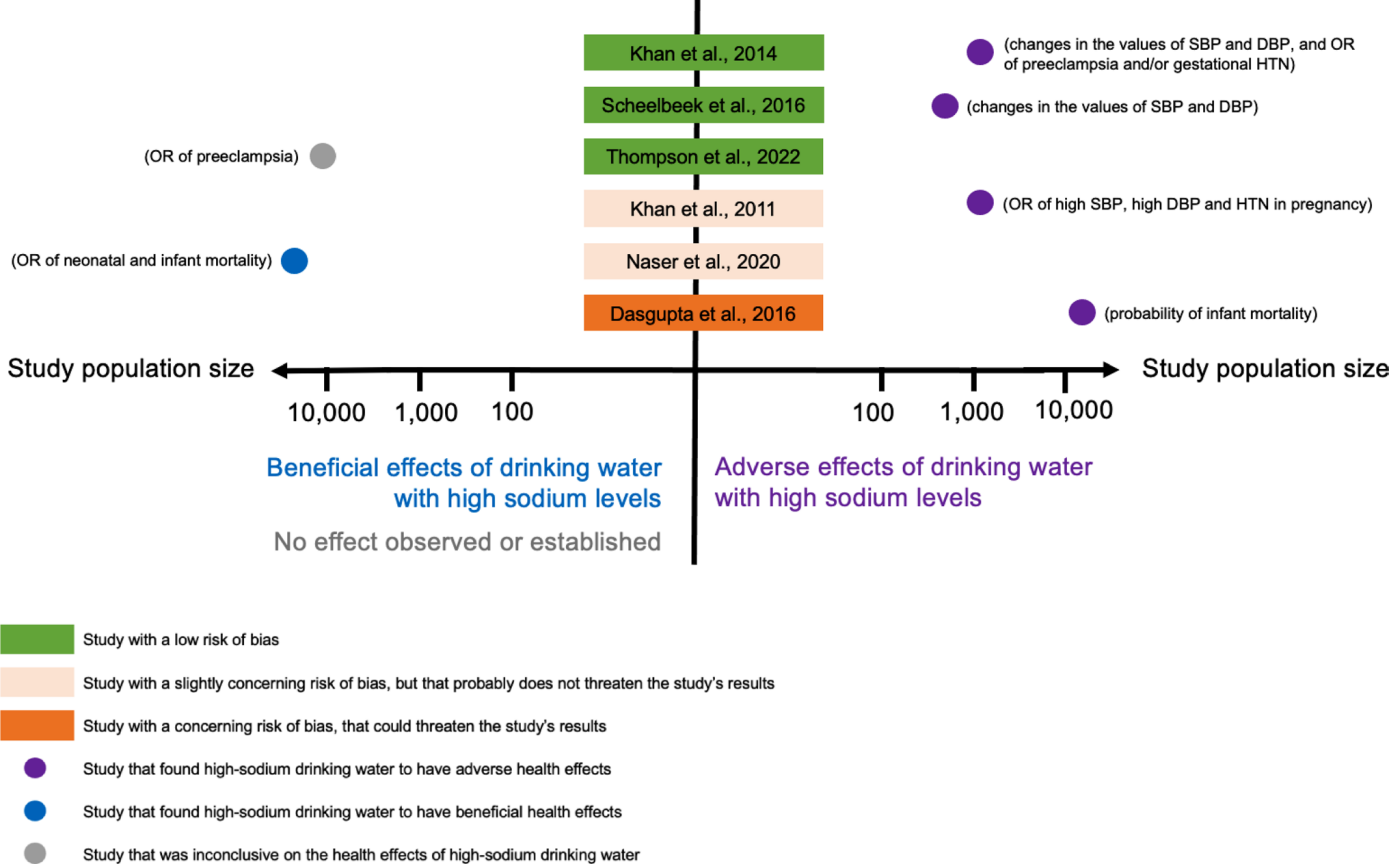
Overall effect on pregnancy-related health outcomes found by each study, weighted by the study’s risk-of-bias score and population size

Table 1 summarises the main findings of the studies. Making a funnel plot was not possible due to heterogeneity. Instead, we plotted the overall effect (beneficial, adverse or inconclusive) of drinking high-sodium water in relation to the study’s risk of bias score and population size (a proxy for the robustness of its findings). The graphs for cardiovascular, renal and pregnancy-related outcomes can be found in Figs. 2, 3 and 4, respectively.

#### Cardiovascular health

Of the 15 papers assessing blood pressure and hypertension, seven found evidence for their statistically significant associations with higher sodium levels in drinking water [12, 15, 17, 19, 22, 24, 40]. Five of these studies were at low risk of bias [12, 15, 21, 23, 26], and two presented some concerns of bias [28, 50]. For example, Al-Nahian et al. [26] reported an odds ratio (OR) of 1.17, with a 95% confidence interval (95%CI) between 0.97 and 1.42 for hypertension in individuals who drank slightly-saline water (1,000–2,000 mg/l, equivalent to 400–800 mgNa/l), and an OR of 1.42 (95%CI: 1.05, 1.91) for those who drank moderately-saline water (> 2,000 mg/l, equivalent to > 800 mgNa/l), compared to those who drank freshwater (< 1,000 mg/l, equivalent to < 400 mgNa/l).

On the other hand, two low risk of bias studies [22, 25] found high salinity levels to have a beneficial effect on blood pressure and hypertension. Naser et al. [22], for example, observed higher systolic blood pressure (+ 2.15 mmHg; 95%CI: +1.02, + 3.27) and diastolic blood pressure (+ 1.82 mmHg; 95%CI: +1.19, + 2.45) in individuals consuming rainwater with low sodium levels (2 mgNa/l), compared to those consuming groundwater with higher sodium levels (312 mgNa/l).

Some studies examined systolic and diastolic blood pressure separately. Naser et al. [25] and Pomeranz et al. [50], for instance, observed that water sodium levels were associated with diastolic but not systolic blood pressure.

Finally, seven studies [24, 45–49, 51] could not establish with certainty any effect, either beneficial or adverse, of drinking water sodium on cardiovascular health outcomes. In a binary comparison of higher-salinity (> 172 mgNa/l) versus lower-salinity (< 172 mgNa/l) water, with low risk of bias, Chakraborty et al. [24] found significantly higher prevalence of hospital visits for cardiovascular disease in the high-salinity group as compared to the low-salinity group (10.1% vs. 2.6%). However, when quartiles of salinity were considered, differences across quartiles were not significant.

Overall, high sodium levels in drinking water were associated with adverse cardiovascular outcomes in most [12, 15, 21, 23, 26, 28, 50], but not all, studies, and the quality of evidence, overall, was moderate.

#### Kidney diseases

All three studies [15, 21, 22] on kidney disease had low risks of bias, and all pointed towards a negative association between renal function with drinking water salinity. Shuvo et al. [21] found relative risk ratios of kidney disease of 2.89 (standard error: 1.28) among consumers of shallow tube-well water (high salinity: 640–866 mgNa/l) and of 4.47 (standard error: 2.01) among consumers of pond water (high salinity: 760–940 mgNa/l), when compared to the consumers of deep tube-well water (low salinity: 400–538 mgNa/l). Similar associations were found by Rosinger et al. [15]: the OR of hyperdiluted urine was 1.34 (95%CI: 0.97, 1.84) for every 100 mgNa/l increase in sodium concentration. Naser et al. [22] had mixed findings. The median 24-hour urine protein ratio was 1.17 (95%CI: 1.02, 1.33) among those consuming groundwater (312 mgNa/l) vs. rainwater (2 mgNa/l), suggesting adverse effects of high-salinity water on renal function [18]. However, it was 0.89 (95%CI: 0.74, 1.07) for pond-water consumers (131 mgNa/l) vs. rainwater consumers (2 mgNa/l), suggesting a protective effect of high-salinity water. The latter finding, though, was not statistically significant [22].

#### Maternal and child health

Six studies [9, 11, 13, 27, 29, 44] looked at pregnancy-related health outcomes. Among the four studies assessing blood pressure, risk of preeclampsia, and gestational hypertension, all [9, 11, 13, 29] found significantly higher risk in women consuming higher-salinity water. With some concerns over bias, Khan et al. [9] reported an OR of 2.39 (95%CI: 1.43, 3.99) for hypertension in pregnancy in the dry vs. the rainy season (daily sodium intake from drinking water of 5–16 g vs. 1 gram). Scheelbeek et al. [11] and Khan et al. [13] reported similar results. Findings of Thompson et al. [29] were less consistent. Consumption of drinking water with higher sodium concentrations (20–69, 70–102, and ≥256 mgNa/l groups) was significantly associated with a higher risk of preeclampsia (ORs 1.38, 1.05, and 1.16, respectively) than consumption of water with lower sodium concentration (< 20 mgNa/l). However, the risk was lower for those consuming water with a sodium concentration of 103–255 mgNa/l, suggesting there was not a clear dose-response relationship. Among the three studies that assessed systolic and diastolic blood pressure in pregnancy separately, one [9] reported no evidence for association with diastolic blood pressure, and two [11, 13] found that both measurements were higher with consumption of high-salinity water. Supplementary Figs. 2 and 3 show the dose-response relationships derived from the results of the studies that were directly comparable.

Lastly, the two studies [27, 44] focussed on infant mortality provided inconsistent results and were at risk of bias due to inaccurate exposure measurement (e.g., using regional soil salinity data [44] and a national salinity map [27]). Dasgupta et al. [44] found that the probability of death of one-month old female infants of lower SES increased by up to 33.3%, when the mother switched from the 5th to the 95th centile of water salinity during the last month of pregnancy. Statistical significance was not provided. Naser et al. [27] found significantly higher risks of neonatal and infant mortality for freshwater consumption (EC < 0.7 mS/cm), compared to the consumption of mildly saline water drinkers (EC ≥ 0.7 and < 2 mS/cm). The risks of both neonatal and infant mortality were significantly higher in the very high-salinity group ((EC ≥ 10 mS/cm), compared to the freshwater group. To note, the sodium levels classified as “mild-” and “moderate-salinity” in this study were relatively low – analogous levels were often used as comparators in other studies (i.e., the non-exposed group).

Overall, higher sodium concentrations in drinking water are linked to elevated blood pressure in pregnancy, higher risks of gestational hypertension, and (with inconsistent results) increased child mortality. The evidence on maternal and child health was of mixed quality and strength.

### Risk of bias, quality and strength of the evidence

Supplementary Table 2 details the risk of bias appraisal of all the studies following the ROBINS-E [43] Cochrane tool. Most studies (*n* = 19) [11–13, 15, 21–29, 44–48, 51] adjusted for important confounders, although only five studies [22, 23, 46, 47, 51] adjusted for dietary sodium intake (cooking and/or table salt), and none adjusted for the natural presence of sodium in food. Three studies [9, 49, 50] did not adjust for any confounders. Overall, 13 studies were found to have a low risk of bias [11–13, 15, 21–26, 29, 48, 51]; nine studies raised some concerns about bias [9, 27, 28, 44–47, 49, 50], with six [28, 44, 45, 47, 49, 50] considered to be of poor quality to the extent that it may have impacted their results.

Supplementary Table 3 presents assessment of the quality and strength of evidence across the studies by outcome type, following the Navigation Guide methodology [52]. The quality and strength of evidence on cardiovascular outcomes was found to be moderate, due to risk of bias, inconsistency and imprecision. The quality of evidence on renal outcomes was good, but with it was of inadequate strength due to the limited number of studies and inconsistency amongst them. Evidence on maternal outcomes was of moderate quality and strength, due to risk of bias, inconsistency, and risk of publication bias.

Although all studies were observational and water salinity measurements could only capture exposure at a specific point of time, the authors assumed chronic exposure as participants used the water sources on a prolonged and regular basis. The “snapshot” measurements of water sodium levels at the time of the studies were used as estimates of the long-term exposure, as has been done in other observational settings [53]. It is important to recognise that switching between sources may have happened, and that the cumulative exposure of each individual depends on the duration of residency in the affected area. This may not have been accounted for in these studies.

## Discussion

### Salinity and health risks

This is the first systematic review evaluating the evidence on the impacts of drinking high-salinity water on cardiovascular, renal, and maternal health. 14 analyses from eight studies at low bias risk [11–13, 15, 21–23, 26] and four studies at moderate bias risk [9, 28, 44, 50] found a higher risk of increased blood pressure, hypertension, renal dysfunction, gestational hypertension, preeclampsia and infant mortality associated with excessive salinity in drinking water. Diastolic blood pressure was less commonly associated with water salinity than systolic blood pressure [9, 25, 50], although both values followed the same trend in most studies [11–13, 15, 22, 23]. Eight studies could not establish with certainty any effect, whether beneficial or adverse [24, 29, 45–49, 51]. Finally, some studies observed worse health outcomes (including blood pressure, gestational hypertension and infant mortality) linked to drinking water with very low salinity [22, 25, 27]. However, it is important to note that one of these studies raised concerns about bias, while another measured electrical conductivity rather than specifically measuring sodium, which complicates the distinction between the effects of different minerals.

Our systematic review has several strengths. We adhered closely to the PRISMA checklist [38], conducted a thorough screening process, and systematically searched five major databases. We used the Cochrane tool ROBINS-E framework [43] for risk of bias appraisal and the Navigation Guide [52] to assess the quality and strength of the body of evidence. However, there are also some limitations. One of the main difficulties was the heterogeneity in the definitions of what constitutes high vs. low water salinity across the studies we reviewed. In addition, by focusing solely on sodium, we may have underestimated the confounding effect of other, beneficial ions, often present in highly mineral water. In terms of study location, a significant portion of the studies were conducted in Bangladesh and the United States, which may limit the generalizability of our findings. The great heterogeneity in methods and reporting of results did not allow for a meta-analysis, preventing the calculation of any summary association measure. We also noted a limited number of larger studies with negative findings, in addition to several smaller studies with positive findings, which suggests publication bias. Due to significant disparities in health outcomes and sodium concentrations, as well as the inability to conduct a meta-analysis or create a funnel plot, our review was unable to determine a health-based threshold for sodium levels in drinking water.

Our findings are consistent with the limited existing reviews on this topic. A systematic review and meta-analysis focusing solely on hypertension found a positive association between drinking water sodium and blood pressure. However, in contrast to our findings, diastolic blood pressure was more strongly associated with water salinity than systolic blood pressure [19]. A narrative (non-systematic) literature review, which broadly assessed the health effects of high-salinity water consumption, linked excessive sodium levels to high blood pressure, cardiovascular morbidity and mortality, preeclampsia, intrauterine growth retardation, preterm birth, maternal and prenatal death, kidney disease, decreased liver function, decreased platelet count, and cholera [54]. A scoping review evaluating the effect of salts (sodium, magnesium, calcium and potassium) in drinking water had mixed findings, with just over half the papers suggesting a detrimental effect of high salinity on blood pressure and hypertension. The other half suggested a beneficial effect of high-salinity water, perhaps due to the presence of magnesium, calcium and potassium, which can have cardioprotective effects [20]. It should be noted that this review was focusing on the overall salt content of water, including salubrious ions, and not solely on sodium as ours did. Our systematic review encompassed several studies that were also included in the three reviews mentioned above. However, our review presents a unique perspective as we focused on three specific health domains and defined “exposure” as sodium concentration above 200 mgNa/l (a higher cut-off than what some reviews have used). In concordance to the previous reviews’ findings, we also observed adverse outcomes associated with drinking water with very low sodium levels. This resonates with previously published material highlighting the risks of drinking overly demineralised water, which is deprived of its beneficial minerals such as magnesium, calcium and potassium [16–18].

To assess the plausibility of a causal relationship – whether drinking highly-saline water causes adverse health outcomes – we used the Bradford-Hill criteria [55]. Our review demonstrates evidence for most criteria. The biological gradient was demonstrated in seven [11–13, 15, 23, 26, 29] out of 13 low bias risk studies, in the form of a dose-response trend, with progressively worse health outcomes linked to higher water sodium levels in water. Consistency was demonstrated by the reproducibility of the findings across different populations and countries. Most papers adjusted for confounders, which strengthens the assumption that the observed outcomes are specific to high-sodium water exposure, although the effects of other minerals present in water are hard to adjust for.

Although most studies were cross-sectional, temporal relationships could be inferred by using transient measurements (drinking water sodium levels at the time of the study) as proxies for historical environmental exposure [53]. Switching of water sources can happen in a participant’s lifetime, and was not always accounted for. Therefore, the studies’ conclusions do not completely satisfy the criterion of temporal sequence, though it is unlikely that people with higher blood pressure or worse renal function choose more saline water. However, one cohort study [26] provided evidence of a temporal relationship: water salinity increased over time, and so did the prevalence of high blood pressure. Regarding biological plausibility, a causal relationship appears realistic, given that the adverse health effects of excessive dietary sodium are already well-established [6] from studies on diet.

The strength of the association varied greatly, ranging from strongly elevated ORs or β-coefficients for adverse outcomes in eight low bias risk studies [11–13, 15, 21–23, 26], to beneficial effects of high-salinity water in two low bias risk studies (which, however, did not provide statistical significance) [22, 25]. Reversibility was demonstrated in one of these studies, which showed that for each 100 mg/L reduction in sodium in drinking water, systolic/diastolic blood pressure was lower on average by 0.95/0.57 mmHg, and odds of hypertension were lower by 14% [23]. Finally, consistency across animal studies and human observations supports the coherence of the relationship between sodium intake and adverse health outcomes. In animal studies, such as those using rats, a consistent and strong association has been shown between high-sodium drinking water and the development of high blood pressure [56, 57]. These studies illustrate how sodium intake via drinking water can trigger mechanisms like fluid retention, sympathetic nervous system activation, and endothelial dysfunction, all contributing to increased blood pressure.

Overall, a causal link between drinking high-sodium water and poor health appears plausible, but more research is needed to establish a clear dose-response relationship with adverse health outcomes. Long-term cohort studies and studies assessing the reversibility of health outcomes following the cessation of high-sodium water consumption would be particularly valuable, such as randomized controlled trials that sequentially provide low-sodium potable water to different communities or households. Future research should ensure reliable exposure assessment, including water samples taken directly at the point of use for each participant and accurate estimation of individual daily water consumption for drinking and cooking. It should also ensure robust outcome measurements, using standardised methods or instruments, and blinding of the assessors whenever possible. Important potential confounders to adjust for include age, sex, body mass index, smoking and use of other stimulants, physical activity, dietary sodium intake, consumption of coffee or tea, family history, personal medical history, medication history, socio-economic status, length of residency in the affected area, ambient temperature at the time of blood pressure measurements, and the presence of other minerals in the water. As a cut-off for “high-salinity” water, we would recommend adhering to the WHO (palatability-based) value of 200 mgNa/l for the time being, until a health-based threshold can be established. Beyond the health outcomes investigated here, future studies should also explore potential effects on skin, liver, blood and infectious diseases. Moreover, an improved understanding of sodium metabolism when ingested through liquids versus solids would facilitate a more accurate interpretation of the findings. Once sufficient primary evidence is available, a quantitative meta-analysis would substantially strengthen our understanding and help define an optimal range of sodium concentration in drinking water. To provide a more holistic perspective, future investigations should also assess the broader community impacts of high-salinity exposure, given evidence linking highly saline water to persistent poverty [58, 59] and lower academic achievement in schoolchildren [59].

### Policy implications

A health-based standard for salinity should be established by a recognised organisation, such as the WHO, to provide policymakers with a clear benchmark for action, in order to improve public health and prevent disease, especially in the most vulnerable communities [5, 60]. The WHO’s dietary guideline assumes that drinking water contributes only a small fraction of daily sodium intake, implying a health-based threshold of approximately 20 mg/L [8]. However, this may not be a practical universal standard, as natural sodium concentrations often exceed this level – not only in coastal areas affected by salinity intrusion but also in regions with high-fluoride groundwater, geothermal springs, and inland aquifers. Despite these challenges, the absence of a health-based benchmark leaves policymakers without a clear reference point for action. Establishing at least a recommended upper limit – even if regionally adaptable – would provide a framework for risk assessment, mitigation efforts, and long-term water safety strategies. Such a guideline should consider both the cumulative sodium intake from all sources and the feasibility of reducing drinking water sodium in different settings, ensuring that public health remains a priority while acknowledging local constraints.

The climate crisis is expected to worsen the issue, as rising sea levels, cyclones, and storm surges contribute to increased salinity in freshwater sources^,^ [2, 61]. Particularly in vulnerable zones, a growing number of people will face chronic exposure to high-salinity water [62]. While the long-term health consequences for the millions of people concerned remain to be seen, they are likely to be significant.

Figure 1 An illustration of the hypothesised process that this study aims to assess. Climate change causes more frequent storms, cyclones and droughts, and leads to sea level rise and altered rainfall patterns, all of which can cause freshwater resources of affected areas to become increasingly saline. Consumption of this water could expose communities to increased amounts of sodium that are beyond the daily recommended intake. This, in turn, could lead to diseases known to be linked to high sodium intake, such as cardiovascular diseases, renal diseases, and adverse maternal and infant outcomes. However, highly-saline water is often rich in other, cardioprotective ions such as calcium, magnesium and potassium, which could (at least partly) mitigate against the adverse effects of sodium.

**Table 1:** Main findings of the studies included

Study		Main findings			
*Studies looking at cardiovascular-related health outcomes*					
Tuthill et al., 1980 [45]	United States	♣ In the Texhoma study: o There were no significant changes in either SBP (98.5–99.0 mmHg in the high-salinity group, versus 98.4–98.5 mmHg in the low-salinity group) or DBP (54.0-57.8 mmHg in the high-salinity group, versus 55.9–57.5 mmHg in the low-salinity group). P-values > 0.05 apart for the male DBP comparison (*p* = 0.09). o There was a statistically significant difference in SBP between the HH group (102.2 mmHg for males and 102.3 mmHg for females) and the HL group (95.1 mmHg for males, *p* = 0.00, and 97.5 mmHg for females; *p* = 0.01) as well as the LL group (97.4 mmHg for males, *p* = 0.02, and 94.7 mmHg for females, *p* = 0.00). o There were no significant changes in SBP between the HL and the LL groups, nor any changes in DBP in any comparison. P-values > 0.05. ♣ In the Ohio water softener study: o The BPs of the higher-salinity groups (“Town Softener”: 102.9/59.9 mmHg for males and 103.4/64.0 mmHg for females; “Military”: 99.4/59.3 mmHg for males and 99.4/64.2 mmHg for females) were not significantly different from the lower-salinity groups (“Town Distribution”: 104.1/60.0 mmHg for males and 98.8/63.9 mmHg for females: “Next City”: 98.3/57.0 mmHg for males and 95.8/63.5 mmHg for females). P-values not given.			
Hallenbeck et al., 1981 [46]	United States	♣ There was no significant difference in SBP (107–115 mmHg) or DBP (68–70 mmHg) in children from the high-salinity community compared to the children from the low-salinity community (SBP 106–119 mmHg; DBP 66–68 mmHg) (p-values > 0.05 in all but one comparisons).			
Armstrong et al., 1982 [51]	Australia	♣ There was no discernible pattern in SBP or DBP in children from towns with varying salinity. The lowest-salinity town (34 mg/l) had a mean SBP of 100 mmHg for boys and 103 mmHg for girls, whereas the highest-salinity town (223 mg/l) had a mean SBP of 98 mmHg for boys and 96 mmHg for girls. The lowest-salinity town had a mean DBP of 47 mmHg for boys and 51 mmHg for girls, whereas the highest-salinity town had a mean DBP of 49 mmHg for boys and 52 mmHg for girls.			
Faust, 1982 [47]	United States	♣ There was no relationship between either SBP, DBP or MAP and intake of sodium from drinking water (whether it be as the proportion of total sodium coming from water as opposed to diet, or as the cumulative exposure to high-sodium water based on the years of residency in the area). Values not given. ♣ There was no change in the mean water sodium concentrations in different BP categories: 184 mgNa/l for the normal BP category: 175 mgNa/l for the borderline BP category (> 140/60 mmHg); and 138 mgNa/l for the high BP category (> 160/69 mmHg). *P* = 0.508.			
Pomrehn et al., 1983 [48]	United States	♣ There was no significant difference in SBP (98.4–99.0 mmHg) or DBP (61.5–64.6 mmHg) in children from high-salinity communities compared to children from low-salinity communities (SBP 97.2-100.3 mmHg; DBP 60.5–64.0 mmHg) (*p* = 0.02).			
Welty et al., 1986 [49]	United States	♣ No sodium concentration level was consistently associated with BP. Sodium levels had either no effect on BP (*p* > 0.05), or were negatively associated with DBP in White non-Hispanic and Hispanic communities (*p* < 0.05). ♣ The prevalence of HTN did not vary between Papago Indians (exposed to high salinity), at 19.5 ± 5.1% for men and 24.7 ± 5.1% for women, and non-Hispanic Whites (not exposed to high-salinity), at 22.5 ± 2.3% for men and 23.8 ± 5.5% for women (not statistically significant). ♣ The standardized mortality rates for HTN-related causes of death were increased for Gila Bend Whites (proportionate mortality rate 8.6) compared to Arizona Whites (proportionate mortality rate 4.1). P-values not given.			
Pomeranz et al., 2000 [50]	Israel	♣ SBP was significantly higher in the high sodium group compared to the low sodium groups, independently from nitrates’ effect: o group 1 (high sodium, high nitrates): 116 ± 12 mmHg; o group 2 (low sodium, high nitrates): 111 ± 11 mmHg (*p* < 0.05 vs. group 1); o group 3 (low sodium, low nitrates): 107 ± 8 mmHg (*p* < 0.05 vs. group 1 and 2). ♣ DBP did not change between low- and high-sodium groups (p-values not given): o group 1: 66 ± 7 mmHg; o group 2: 66 ± 6 mmHg; o group 3: 63 ± 8 mmHg. ♣ MAP was significantly higher in the high-sodium group compared to the low-sodium groups, independently from nitrates’ effect: o group 1: 86 ± 9 mmHg; o group 2: 83 ± 8 mmHg (*p* < 0.05 vs. group 1); o group 3: 81 ± 7 mmHg (*p* < 0.05 vs. group 1).			
Talukder et al., 2016 [12]	Bangladesh	♣ The β-coefficient for higher SBP is 3.46 (95%CI: 0.75, 6.17; *p* = 0.01) and for higher DBP is 2.77 (95%CI: 0.31, 5.24; *p* = 0.03) in the high-salinity group compared to the low-salinity group.			
Scheelbeek et al., 2017 [23]	Bangladesh	♣ For each 100 mg/l decrease of sodium in the drinking water, SBP decreased by 0.95 (95%CI: 0.71, 1.20; *p* < 0.001) mmHg and DBP decreased by 0.57 (95%CI: 0.38, 0.76; *p* < 0.001) mmHg. ♣ For each 100 mg/l decrease of sodium in the drinking water, the mixed logistic regression models for HTN found an OR of 0.862 (95%CI: 0.79, 0.93; *p* < 0.001). ♣ Between-year comparison: those whose water sodium concentration increased saw their BP increasing, and vice-versa (significant - data not given).			
Talukder et al., 2018 [28]	Vietnam	♣ Compared to not saline-exposed municipalities, the OR for hospital admission for HTN was 1.09 (95%CI: 1.03, 1.14) in salinity-exposed area. *P* = 0.001.			
Al Nahian et al., 2018 [26]	Bangladesh	♣ The OR of HTN in slightly saline drinkers compared to freshwater drinkers was 1.17 (95%CI: 0.97, 1.42; *p* < 0.1). ♣ The OR of HTN in moderately saline drinkers compared to freshwater drinkers was 1.42 (95%CI: 1.05, 1.91; *p* < 0.05). ♣ In those < 35 years old, 34.3% had high BP among freshwater drinkers, 42.6% among moderate salinity water drinkers. P-values not given. ♣ In those > 35 years old, 47.8% had high BP among freshwater drinkers, 53.8% among slightly saline water drinkers, and 62.5% among moderately saline drinkers. P-values not given. ♣ The prevalence of high BP increased from one round to the other as the salinity of the water increased: for freshwater drinkers, from 46.6% (round 1) to 49% (round 3); for moderate-salinity water drinkers, from 58.6% (round 1) to 68.6% (round 3). P-values not given.			
Chakraborty et al., 2019 [24]	Bangladesh	♣ The high-salinity group had significantly higher prevalence of hospital visits for CVD (10.1%) than the low-salinity group (2.6%). *P* < 0.05. ♣ Compared to low-salinity quartile, the ORs of hospital visits for CVD non-significantly increased for higher salinity quartiles: OR 1.64 (95%CI: 0.21, 6.12) for Q2 and OR 1.64 (95%CI: 0.25, 10.99) for Q4. P-value not given. ♣ Compared to low-salinity quartile, the ORs of hospital visits for CVD non-significantly decreased for higher salinity quartile: OR 0.13 (95%CI: 0.01, 1.11) for Q3. P-value not given.			
Naser et al., 2019 [25]	Bangladesh	♣ Compared to freshwater, the OR of elevated BP was: 0.92 (95%CI: 0.71, 1.18) for mild-salinity water; 0.96 (95%CI: 0.71, 1.30) for moderate-salinity water. ♣ Compared to freshwater, the OR of stage 1 HTN was: 0.60 (95%CI: 0.43, 0.84) for mild-salinity water; 0.77 (95%CI: 0.51, 1.17) for moderate-salinity water. ♣ Compared to freshwater, the OR of stage 2 HTN was: 0.56 (95%CI: 0.46, 0.89) for mild-salinity water; 0.61 (95%CI: 0.35, 1.09) for moderate-salinity water. P-values not given. ♣ The propensity score matched analyses for the association of low versus high water EC distribution on BP was of −1.64 (95%CI: −3.16, −0.12) for SBP and of −1.54 (95%CI: −2.52, −0.58). ♣ One SD increase in urinary sodium excretion caused an increase in SBP (+ 0.48 mmHg; 95%CI: +0.14, + 0.81). P-value not given. ♣ One SD increase in urinary sodium excretion caused no change in DBP (+ 0.00 mmHg; 95%CI: −0.20, + 0.20). P-value not given.			
*Studies looking at kidney-related and cardiovascular-related health outcomes*					
Shuvo et al., 2020 [21]	Bangladesh	♣ Compared to deep tube-well water, the RRR of HTN: o for shallow tube-well water was 3.12 (SE: 1.46; *p* < 0.01); o for pond water was 3.98 (SE: 1.24; *p* < 0.01). ♣ Compared to deep tube-well water, the RRR of kidney disease: o for shallow tube-well water was 2.98 (SE: 1.28; *p* < 0.05); o for pond water was 4.47 (SE: 2.01; *p* < 0.01).			
Naser et al., 2020 [22]	Bangladesh	♣ Compared to coastal groundwater drinkers, rainwater drinkers had (p-value not given): o 2.15 (95%CI: 1.02, 3.27) mmHg higher SBP; o 1.82 (95%CI: 1.19, 2.45) mmHg higher DBP. ♣ Compared to pond water drinkers, rainwater drinkers had (p-value not given): o 0.91 (95%CI: −0.54, 2.36) mmHg higher SBP; o 0.53 (95%CI: −0.39, 1.44) mmHg higher DBP. ♣ The median 24-hour urine protein ratio was 1.17 (95%CI: 1.02, 1.33) for coastal groundwater drinkers compared to rainwater drinkers. P-value not given. ♣ The median 24-hour urine protein ratio was 0.89 (95%CI: 0.74, 1.07) for pond water drinkers compared to rainwater drinkers. P-value not given.			
Rosinger et al., 2021 [15]	Kenya	♣ For every 100 mg/l increase in sodium concentration of the drinking water: o increase in HTN (OR 1.45, 95%CI: 1.09, 1.94; *p* < 0.05); o increase in SBP (β coefficient 0.81; 95%CI: −0.93, 2.54; p-value not given); o increase in DBP (β coefficient 1.15; 95%CI: −0.03, 2.33; *p* < 0.1). ♣ Positive association with hyperdilute urine (OR 1.34; 95%CI: 0.97, 1.84; *p* < 0.1) for every 100 mg/l increase in sodium concentration.			
*Studies looking at pregnancy-related health outcomes*					
Khan et al., 2011 [9]	Bangladesh	♣ Non-significant (*p* = 0.53) increasing association between quartiles of urinary sodium (compared to urinary sodium < 105 mmol/day) and SBP > 130 mmHg: o OR 1.54 (95%CI: 0.37, 6.93) for urinary sodium 105–155 mmol/day; o OR 1.42 (95%CI: 0.34, 5.87) for urinary sodium 155–204 mmol/day; o OR 2.41 (95%CI: 0.64, 9.13) for urinary sodium > 204 mmol/day. ♣ Urinary sodium was not associated with DBP > 85 mmHg. P-value not given. ♣ OR for HTN in pregnancy during the dry season is 2.39 (95%CI: 1.43, 3.99) compared to the rain season.			
Khan et al., 2014 [13]	Bangladesh	♣ Mean SBP/DBP (in mmHg) significantly increased as the salinity of the sources increased: 102.4 ± 16.2/66.2 ± 12.0 for rainwater; 112.7 ± 22.7/73.2 ± 14.8 for filtered and unfiltered pond water; 119.4 ± 26.7/76.1 ± 17.3 for tube-well water. *P* < 0.001. ♣ Compared to rainwater, the OR of preeclampsia and/or gestational HTN was 5.32 (95%CI: 2.41, 11.7) for filtered pond water, 5.31 (95%CI: 2.60, 10.9) for unfiltered pond water, 8.30 (95%CI: 4.20, 16.4) for tube-well water. All p-values < 0.001. ♣ Compared to water with sodium concentration < 300 mg/l, the OR of preeclampsia and/or gestational HTN was 3.30 (95%CI: 2.00, 5.51) for the 300–600 mgNa/l group, 4.40 (95%CI: 2.70, 7.25) for the 600–900 mgNa/l group, 5.48 (95%CI: 3.30, 9.11) for the > 900 mgNa/l group. All p-values < 0.001.			
Scheelbeek et al., 2016 [11]	Bangladesh	♣ Compared to rainwater, o those drinking pond water had SBP + 3.62 (95%CI: 1.20, 6.04) mmHg and DBP + 1.72 (95%CI: −0.36, 3.80) mmHg; o those drinking tube well water had SBP + 4.85 (95%CI: 2.55, 7.25) mmHg and DBP + 2.30 (95%CI: 0.33, 4.23) mmHg. ♣ The mean BP increased with water sources of higher salinity. It was 100/65 for rainwater, 105/70 for pond water, and 110/70 for tube well water. P-value not given. ♣ Compared to rainwater, the mixed linear regression model showed a difference in SBP of 3.62 (95%CI: 1.20, 6.04; *p* = 0.003) mmHg for pond water, and 4.85 (95%CI: 2.55, 7.15; *p* < 0.001) mmHg for tube well water. ♣ Compared to rainwater, the mixed linear regression model showed a difference in DBP of 1.72 (95%CI: −0.36, 3.80; *p* = 0.105) mmHg for pond water, and 2.30 (95%CI: 0.33, 4.23; *p* = 0.022) mmHg for tube well water.			
Dasgupta et al., 2016 [44]	Bangladesh	♣ The probability of mortality of a model child increases by 33.3% (if maternal age 30) and by 29.1% (if maternal age 17) if water salinity consumed during the last month of pregnancy increases from the 5th to the 95th percentile. P-value not given.			
Naser et al., 2020 [27]	Bangladesh	♣ The ORs of neonatal and infant deaths first decreased from, respectively, 5.1 (95%CI: 4.7, 5.6) and 6.0 (95%CI: 5.6, 6.6) for freshwater drinkers, to 3.5 (95%CI: 2.9, 4.3) and 4.0 (95%CI: 3.3, 4.8) for mild-salinity water drinkers. All p-values < 0.001. ♣ The ORs of neonatal and infant deaths subsequently increased again: respectively, 3.4 (95%CI: 2.8, 4.1) and 4.5 (95%CI: 3.8, 5.2) for moderate-salinity water drinkers (all p-values < 0.001), and further to 5.1 (95%CI: 3.8, 6.7; *p* = 0.984) and 6.5 (95%CI: 5.1, 8.1; *p* = 0.606) for severe-salinity water drinkers.			
Thompson et al., 2022 [29]	United States	♣ Compared to water sodium < 20 mg/l, the OR of preeclampsia for those drinking water with sodium concentration 20–69 mg/l is 1.38 (95%CI: 1.13, 1.69). *P* = 0.002. ♣ Compared to water sodium < 20 mg/l, the risk of preeclampsia increased for sodium concentrations 70–102 mg/l (OR 1.05; 95%CI: 0.83, 1.32; *p* = 0.706) and ≥ 256 mg/l (OR 1.16; 95%CI: 0.93, 1.44; *p* = 0.180). ♣ Compared to water sodium < 20 mg/l, the risk of preeclampsia decreased for sodium concentration 103–255 mg/l (OR 0.88; 95%CI: 0.69, 1.13; *p* = 0.322).			
*mmol/day = millimoles per day* *mg/l = milligrams per litre* *mgNa/l = milligrams of sodium per litre* *mmHg = millimetres of mercury*			*BP = blood pressure* *SBP = systolic blood pressure* *DBP = diastolic blood pressure* *MAP = mean arterial pressure*	*HTN = hypertension* *CVD = cardiovascular disease* *OR = odds ratio* *RRR = relative risk ratio*	*± = SD = standard deviation* *95%CI = 95% confidence interval* *SE = standard error* *EC = electrical conductivity*

Table 1 A table giving the main numerical findings with the confidence intervals and statistical significance of each of the studies included. The studies are arranged into three categories depending on the type of health outcomes they look at: cardiovascular-related, kidney-related and pregnancy-related. All the findings shown here are the model that adjusted to the most variables, when there were several models accounting for confounders.

Figure 2 Although plotting a funnel plot was not possible due to heterogeneity in the studies’ exposures and outcomes, we summarised the overall findings by dividing them into a binary outcome: beneficial or adverse effects (on cardiovascular outcomes) of drinking water with high sodium levels. Studies were listed based on their risk of bias assessment, with lower risk studies at the top. The strength of the findings was weighted by the study’s population size. To put the effects into context, we indicated in parenthesis next to each study what the outcome assessed was. The graph shows studies of all sizes and of all risk of bias scores being published, regardless of whether the result was positive, negative or null. This suggests there is no publication bias on this health outcome.

Figure 3 Although plotting a funnel plot was not possible due to heterogeneity in the studies’ exposures and outcomes, we summarised the overall findings by dividing them into a binary outcome: beneficial or adverse effects (on kidney-related health outcomes) of drinking water with high sodium levels. Studies were listed based on their risk-of-bias assessment, with lower risk studies at the top. The strength of the findings was weighted by the study’s population size. To put the effects into context, we indicated in parenthesis next to each study what the outcome assessed was. Only three studies on this health outcome have been published; all were at low risk of bias and all showed high-salinity water to have adverse effects on renal function. It is difficult to comment on possible publication bias with such a limited sample.

Figure 4 Although plotting a funnel plot was not possible due to heterogeneity in the studies’ exposures and outcomes, we summarised the overall findings by dividing them into a binary outcome: beneficial or adverse effects (on kidney-related health outcomes) of drinking water with high sodium levels. Studies were listed based on their risk-of-bias assessment, with lower risk studies at the top. The strength of the findings was weighted by the study’s population size. To put the effects into context, we indicated in parenthesis next to each study what the outcome assessed was. The graph raises concerns about a possible publication bias, as two large studies were inconclusive or had negative findings, whereas studies with smaller populations had positive findings.

## Supplementary Information

Below is the link to the electronic supplementary material.

Supplementary material 1 (DOCX 565.0 kb)
