# Supplementary material for: Adverse health outcomes associated with drinking highly saline water: a systematic review
Source: Eur J Epidemiol. 2025 Sep 29;40(11):1307–22. doi: 10.1007/s10654-025-01307-9 (PMC12696076; doi:10.1007/s10654-025-01307-9)
Supplement: Supplementary file 1 — Supplementary material 1 (DOCX 565.0 kb) [file 10654_2025_1307_MOESM1_ESM.docx]

**SUPPLEMENTARY INFORMATION**


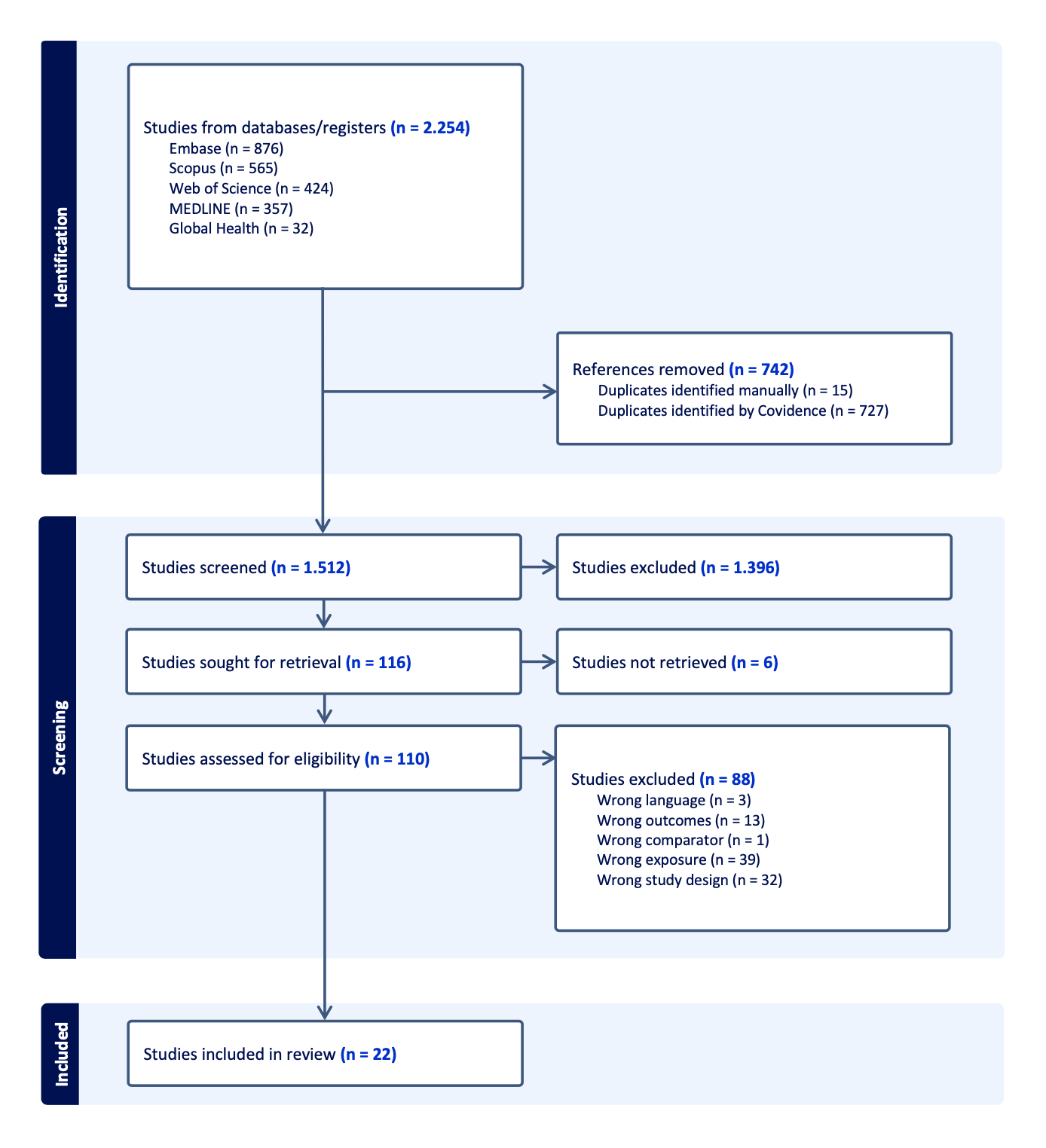


Supplementary figure 1. PRISMA [38] diagram of the screening process.

*Supplementary figure 1 – PRISMA [38] diagram showing the number of papers included and excluded at each stage of screening, and the reasons for exclusion. The search initially yielded 2.254 studies, of which 22 were kept for the final analysis. The screening process was a double, blinded, screening from two authors, complemented with a random 15% sample screened by a third researcher at each stage of the process. Conflicts were resolved by involving a third party, blinded to the decisions of the previous screeners.*

Supplementary table 1. General characteristics and risk of bias assessment of the studies included.

| **Title** | **Authors & year** | **Study design** | | **Population(s)** | **Exposure(s)** | **Comparator(s)** | | **Outcome(s)** | **Confounders adjusted for** | | **Quality appraisal (ROBINS-E)** |
| --- | --- | --- | --- | --- | --- | --- | --- | --- | --- | --- | --- |
| *Studies looking at cardiovascular-related health outcomes* | | | | | | | | | | | |
| The influence of naturally and artificially elevated levels of sodium in drinking water on blood pressure in school children. | Tuthill, Sonich, Okun & Greathouse, 1980 [45] | Cross-sectional study | | Texhoma study: 177 school children (grade 7) in the United States | 114 children in the high-salinity group (285 mg/l) | 63 children in the low-salinity group (10 mg/l) | | Systolic and diastolic BP | Age, sex. | | Some concerns (uncontrolled confounding). Direction of bias unpredictable. Cannot tell if it could threaten the conclusions. |
|  |  |  |  |  | HH: 97 children consuming high-salinity water both at home (250-300 mg/l) and at school (285 mg/l) | HL: 59 children consuming high-salinity water at home (250-300 mg/l) and low-salinity water at school (10 mg/l) | |  |  |  |  |
|  |  |  |  |  |  | LL: 72 children consuming low-salinity water both at home and at school (10 mg/l) | |  |  |  |  |
|  |  |  |  | Ohio study: 326 school children (grade 7) in the United States | “Town Softener” group (water sodium concentration 7-254 mg/l) and “Military” group (water sodium concentration 175-251 mg/l) | “Town Distribution” group (water sodium concentration 8-27 mg/l) and “Next Town” group (water sodium concentration 19-23 mg/l) | |  |  |  |  |
| High sodium in drinking water and its effect on blood pressure. | Hallenbeck, Brenniman & Anderson, 1981 [46] | Cross-sectional study | | 638 high-school students in the United States | Community with high-salinity public drinking water (varying between 300-700 mg/l, average of 405 mg/l) | Community with low-salinity public drinking water (4 mg/l) | | Systolic and diastolic BP | Age, BMI, smoking, estimated dietary sodium intake, months of residency in the community. | | Some concerns (uncontrolled confounding and selection of participants). Direction of bias towards null hypothesis. No threats to conclusions. |
| Water sodium and blood pressure in rural school children. | Armstrong, Margetts, McCall & Binns, 1982 [51] | Cross-sectional study | | 635 school children (aged 12-13) in Australia | Two towns with municipal water sodium concentrations of 205 and 223 mg/l | Four towns with municipal water sodium concentrations of 34, 51, 109 and 110 mg/l | | Systolic and diastolic BP | Age, sex, physical activity, dietary salt intake, weight, height, skinfold thickness, coffee and tea intake, temperature. | | Low risk of bias (uncontrolled confounding). Direction of bias unpredictable. No threats to conclusions. |
| Effects of drinking water and total sodium intake on blood pressure. | Faust, 1982 [47] | Cross-sectional study | | 295 people (mean age 29.2) in the United States | Streets with municipal water sodium concentrations of 222.0, 466.0, 484.5 and 583.0 mg/l (after softening) | Streets with municipal water sodium concentrations of 137.0, 141.5 and 198.5 mg/l + users of bottled water | | Systolic, diastolic and mean BP + prevalence of borderline BP (> 140/90 mmHg) and high BP (> 160/96 mmHg) | Dietary salt intake, time of residency in the area. | | Some concerns (uncontrolled confounding). Direction of bias unpredictable. Cannot tell if it would threaten the conclusions. |
| Community differences in blood pressure levels and drinking water sodium. | Pomrehn, Clarke, Sowers, Wallace & Lauer, 1983 [48] | Cros-sectional study | | 2164 school children (grades 2-5) and 204 families (two parents and the child) in the United States | Four communities in which the municipal water had concentrations > 200 mg/l | Four communities in which the municipal water had concentrations < 200 mg/l | | Systolic and diastolic BP | Age, sex, weight, heart rate, family history. | | Low risk of bias (uncontrolled confounding). Direction of bias unpredictable. No threats to conclusions. |
| Effects of exposure to salty drinking-water in an Arizona community. | Welty, Freni-Titulaer, Zack, Weber, Sippel, Huete, Justice, Dever & Murphy, 1986 [49] | Cross-sectional study + ecological study | | 717 people aged 0-98 years (72 Papago Indian households and 117 non-Indian households) in the United States | Gila Bend water sources of sodium concentrations ranging 200-408 mg/l | Gila Bend water sources of sodium concentrations < 200 mg/l | | Systolic and diastolic BP | - | | Some concerns (uncontrolled confounding). Direction of bias unpredictable. Cannot tell if it would threaten the conclusions. |
|  |  |  |  |  | Papago Indian community, consuming on average 1.1 gNa/day from drinking water alone | White non-Hispanic and Hispanic communities, consuming on average 0.8 and 0.6 gNa/day from drinking water respectively | | Prevalence of HTN |  |  |  |
|  |  |  |  |  | Death records of Gila Bend White communities, consuming on average 0.6-0.8 gNa/day from drinking water, from 1970 to 1980 | Death records of Arizona White communities in 1979, with presumably lower sodium concentrations in drinking water | | HTN-related mortality |  |  |  |
| Elevated salt and nitrate levels in drinking water cause an increase of blood pressure in schoolchildren. | Pomeranz, Korzets, Vanunu, Krystal & Wolach, 2000 [50] | Cross-sectional study | | 951 school children in Israel | Group 1: high sodium (196 mg/l) and high nitrates (49 mg/l) drinking water | Group 2: low sodium (25 mg/l) and high nitrates (40 mg/l) drinking water + group 3: low sodium (35 mg/l) and low nitrates (15mg/l) drinking water) | | Systolic, diastolic and mean BP | - | | Some concerns (information missing on the data source on water salinity and nitrate levels). Direction of bias unpredictable. Cannot tell if it would threaten the conclusions. |
| The effect of drinking water salinity on blood pressure in young adults of coastal Bangladesh. | Talukder, Rutherford, Phung, Islam & Chu, 2016 [12] | Cross-sectional survey | | 253 young adults, aged 19-25 in Bangladesh | High drinking water salinity group (> 600 mgNa/l) | Low drinking water salinity group (< 600 mgNa/l) | | Systolic and diastolic BP | Age, education, occupation, SES, family history of HTN, BMI, vegetable, fruit and fish intake, and tobacco smoking/chewing. | | Low risk of bias (uncontrolled confounding). Direction of bias unpredictable. No threats to conclusions. |
| Drinking Water Salinity and Raised Blood Pressure: Evidence from a Cohort Study in Coastal Bangladesh. | Scheelbeek, Chowdhury, Haines, Alam, Hoque, Butler, Khan, Mojumder, Blangiardo, Elliott & Vineis, 2017 [23] | Case-control study | | 581 adults in Bangladesh | Higher levels of sodium concentration in drinking water (stratified per 100 mg/l change) | Lower levels of sodium concentration in drinking water (stratified per 100 mg/l change) | | Systolic and diastolic BP + odds of HTN | Age, sex, physical activity, BMI, smoking, demographic factors, SES, environmental and weather exposures, underlying diseases, education, religion, use of local stimulants, exposure to chemicals, and estimated dietary salt intake. | | Low risk of bias. Direction of bias unpredictable. No threats to conclusions. |
| Association between salinity and hospital admission for hypertension: an ecological case-control study in the Mekong Delta Region in Vietnam. | Talukder, Rutherford, Chu, Nguyen & Phung, 2018 [28] | Ecological case-control study | | 573,650 adult hospital admissions, of which 22,382 cases (HTN as primary diagnosis) in Vietnam | Salinity-exposed provinces | 551,668 controls: adults admitted to the same hospitals but not for HTN as a primary diagnosis | | Adjusted ORs of hospital admission for HTN | Age, sex, education, and average household income. | | Some concerns (risk of ecological bias + information missing on the date of water salinity data collection). Direction of bias unpredictable. Cannot tell if it would threaten the conclusions. |
| Drinking water salinity associated health crisis in coastal Bangladesh. | Al Nahian, Ahmed, Lázár, Hutton, Salehin & Streatfield, 2018 [26] | Longitudinal survey | | 1,586, 1,516 and 1,531 households in the first, second and third survey rounds in Bangladesh | Slightly saline (1,000-2,000 mgNa/l) and moderately saline (> 2,000 mgNa/l) drinking water | Freshwater (< 1,000 mgNa/l) | | High BP, defined as being on antihypertensive medication, pre-HTN, or HTN as per BP measurements | Age, sex, religion, education, depth of the tube-well, physical activity, dietary intake, landownership, and SES | | Low risk of bias (uncontrolled confounding). Direction of bias unpredictable. No threats to conclusions. |
| Health Implications of Drinking Water Salinity in Coastal Areas of Bangladesh. | Chakraborty, Khan, Dibaba, Khan, Ahmed & Islam, 2019 [24] | Cross-sectional study | | 157 heads of households in Bangladesh | High water salinity group (> 172 mgNa/l) | Low water salinity group (< 172 mgNa/l) | | Prevalence of households with a member who had 3 or more visits to the hospital because of CVD in the last year | Education, and annual household income. | | Low risk of bias (uncontrolled confounding). Direction of bias unpredictable. No threats to conclusions. |
| Drinking Water Salinity, Urinary Macro-Mineral Excretions, and Blood Pressure in the Southwest Coastal Population of Bangladesh. | Naser, Rahman, Unicomb, Doza, Gazi, Alam, Karim, Uddin, Khan, Ahmed, Shamsudduha, Anand, Narayan, Chang, Luby, Gribble & Clasen, 2019 [25] | Cross-sectional study | | Data from 2 studies: 383 + 1191 participants in Bangladesh | Mild (EC ≥ 0.7 and < 2 mS/cm) and moderate (EC ≥ 2 and < 10 mS/cm) salinity groups | Freshwater group (EC < 0.7 mS/cm) | | Systolic and diastolic BP | Age, sex, BMI, smoking, physical activities, alcohol consumption, consumption of additional table salt with food, sleep hours, religion, and household wealth. | | Low risk of bias. Direction of bias unpredictable. No threats to conclusions. |
| *Studies looking at kidney-related and cardiovascular-related health outcomes* | | | | | | | | | | | |
| Exploring the impact of soil and water salinity on dietary behavior and health risk of coastal communities in Bangladesh. | Shuvo, Zahid, Rahman & Parvin, 2020 [21] | Cross-sectional survey | | 240 adults living in coastal villages, of which some reported having HTN or kidney disease in Bangladesh | Drinking higher salinity water: shallow tube-well (640-866 mgNaCl/l), pond water (760-940 mgNaCl/l) | Drinking lower salinity water: deep tube well (400-538 mgNaCl/l) | | Disease (HTN and kidney disease) as self-reported by participants in the survey | Sex, age, region, source of drinking water, source of the cooking water, and source of bathing water. | | Low risk of bias (uncontrolled confounding). Direction of bias unpredictable. No threats to conclusions. |
| Associations of drinking rainwater with macro-mineral intake and cardiometabolic health: a pooled cohort analysis in Bangladesh, 2016-2019. | Naser, Rahman, Unicomb, Parvez, Islam, Doza, Khan, Ahmed, Anand, Luby, Shamsudduha, Gribble, Narayan & Clasen, 2020 [22] | Case-control study | | 10,030 person-visits (8,261 from coastal region, 1,773 from non-coastal region) in Bangladesh | Drinking coastal groundwater (mean sodium concentration 311.64 mg/l), non-coastal groundwater (13.95 mg/l) or pond water (130.71 mg/l) | Drinking rainwater (mean sodium concentration 2.36 mg/l) | | 24h urinary sodium, SBP, DBP, 24h urinary protein, fasting total cholesterol, HDL-C, fasting triglycerides | Age, sex, BMI, smoking, alcohol consumption, physical activity, religion, sleep hours, consumption of table salt with food, household wealth, and seasonality. | | Low risk of bias. Direction of bias unpredictable. No threats to conclusions. |
| Drinking water salinity is associated with hypertension and hyperdilute urine among Daasanach pastoralists in Northern Kenya. | Rosinger, Bethancourt, Swanson, Nzunza, Saunders, Dhanasekar, Kenney, Hu, Douglass, Ndiema, Braun & Pontzer, 2021 [15] | Cross-sectional study | | 226 non-pregnant adult (> 18 years) Daasanach pastoralists in Kenya | Higher levels of water salinity (per 100 mgNa/l increase) | Lower levels of water salinity (per 100 mgNa/l increase) | | Hyperdilute urine (USG < 1.003 - proxy for renal function and CKD) + BP + prevalence of HTN (> 140/90 mmHg) | Sex, age, BMI, time of day, heat index, milk consumption, tea and coffee consumption, kidney problems, diabetes, household wealth, mobility, and physical activity. | | Low risk of bias (uncontrolled confounding). Direction of bias unpredictable. No threats to conclusions. |
| *Studies looking at pregnancy-related health outcomes* | | | | | | | | | | | |
| Drinking Water Salinity and Maternal Health in Coastal Bangladesh: Implications of Climate Change. | Khan, Ireson, Kovats, Mojumder, Khusru, Rahman, & Vineis, 2011 [9] | Descriptive study | | 343 pregnant women in the community + 969 pregnant women attending the hospital for pregnancy-related complications in Bangladesh | Dry season: 5-16 gNa/day intake from drinking water | Rain season: 1.2 gNa/day intake from drinking water | | High urinary sodium excretion (≥ 100 mmol/day) + high BP (> 130 mmHg systolic or > 85 mmHg diastolic) + hospital-based prevalence of gestational HTN | **-** | | Some concerns (uncontrolled confounding + the salinity data not being contemporary to the study itself). Direction of bias towards null hypothesis. No threats to conclusions. |
|  |  |  |  |  | Shallow groundwater: salinity 0.60-2.60 ppt | Rain and filtered water: negligible salinity levels | |  |  |  |  |
| Salinity in drinking water and the risk of (pre)eclampsia and gestational hypertension in coastal Bangladesh: a case-control study. | Khan, Scheelbeek, Shilpi, Chan, Mojumder, Rahman, Haines & Vineis, 2014 [13] | Case-control study | | Pregnant women in their 20^th^ gestational week or more: 202 cases of (pre)eclampsia or gestational HTN + 1,006 normotensive controls in Bangladesh | High salinity water sources: filtered pond water (410.8 mgNa/l), unfiltered pond water (374.3 mgNa/l) or tube-well water (713. 9 mgNa/l) | Low salinity water source: rainwater (66 mgNa/l) | | Systolic and diastolic BP + ORs of (pre)eclampsia + ORs of gestational HTN | Age, parity, SES, and mid-upper arm circumference. | | Low risk of bias (uncontrolled confounding). Direction of bias unpredictable. No threats to conclusions. |
|  |  |  |  |  | High sodium concentration in drinking water groups (> 300 mgNa/l) | Low sodium concentration in drinking water group (< 300 mgNa/l) | |  |  |  |  |
| Drinking Water Sodium and Elevated Blood Pressure of Healthy Pregnant Women in Salinity-Affected Coastal Areas. | Scheelbeek, Khan, Mojumder, Elliott, & Vineis, 2016 [11] | Case-control study | | 701 women in their 20th week of gestation in Bangladesh | Drinking pond water (median sodium concentration 208 mg/l) or tube well water (704 mg/l) | Drinking rainwater (median sodium concentration 31 mg/l) | | Systolic and diastolic BP | Age, nutritional status, physical activity, parity, local, social, behavioural and environmental factors. | | Low risk of bias (uncontrolled confounding). Direction of bias unpredictable. No threats to conclusions. |
| Drinking Water Salinity and Infant Mortality in Coastal Bangladesh. | Dasgupta, Huq & Wheeler, 2016 [44] | Descriptive study | | 39,150 children in Bangladesh | Drinking water salinity level in the 95th percentile (7.83 dS/m) | Drinking water salinity in the 5th percentile (0.52 dS/m) | | Adjusted mortality probability of children | Child sex, age and education of the mother, sex of household head, household wealth, toilet facilities, water sources, and cooking fuels. | | Some concerns (using soil salinity data as a proxy for water salinity + the missing data filled by calculating averages). Direction of bias unpredictable. Cannot tell if it would threaten the conclusions. |
| Modeling the Relationship of Groundwater Salinity to Neonatal and Infant Mortality From the Bangladesh Demographic Health Survey 2000 to 2014. | Naser, Wang, Shamsudduha, Chellaraj & Joseph, 2020 [27] | Descriptive study | | 185,466 live births + 12,053 neonatal deaths + 16,015 infant deaths recorded in Bangladesh | Mothers drinking water moderate or severe salinity water (mean sodium concentration of 173 and 248 mg/l, respectively) | Mothers drinking freshwater or mild salinity water (mean sodium concentration of 26 and 63 mg/l, respectively) | | Neonatal (within 1 month) and infant (within 1 year) mortality per 100 live births | Maternal age, birth order, child sex, parents’ years of education, rural or urban residence, household wealth score, improved sanitation, child's birth year, maternal marital status, depth of tubewell, geographical division, and river basin. | | Some concerns (uncontrolled confounding + inappropriate methods for measurement of exposure). Direction of bias unpredictable. No threats to conclusions. |
| Sodium concentrations in municipal drinking water are associated with an increased risk of preeclampsia. | Thompson, Cwiertny, Davis, Grant, Land, Landsteiner, Latta, Hunter, Jones, Lehmler, Santillan & Santillan, 2022 [29] | Retrospective case-control study | | 10,114 pregnant women presenting to hospitals and clinics in the United States | Higher concentrations of sodium in drinking water categories (103-255 mgNa/l and > 256 mgNa/l) | Lower concentrations of sodium in municipal water (< 20 mgNa/l) | | Adjusted ORs of preeclampsia | Race, ethnicity, gestational age, parity, newborn count and BMI. | | Low risk of bias (uncontrolled confounding). Direction of bias unpredictable. No threats to conclusions. |
| *mS/cm = millisiemens per centimetre*  *dS/m = decisiemens per metre*  *gNa/day = grams of sodium per day*  *mgNa/l = milligrams of sodium per litre*  *mgNaCl/l = milligrams of sodium chloride per litre*  *mg/l = milligrams per litre* | | | *mmol/day = millimoles per day*  *ppt = parts per thousands*  *mmHg = millimetres of mercury*  *BP = blood pressure*  *SBP = systolic blood pressure*  *DBP = diastolic blood pressure* | | | | *HTN = hypertension*  *CVD = cardiovascular disease*  *CAD = coronary artery disease*  *MI = myocardial infarction*  *IHD = ischaemic heart disease*  *HDL-C = high density lipoprotein – cholesterol* | | | *USG = urine specific gravity*  *CKD = chronic kidney disease*  *BMI = body mass index*  *SES = socio-economic status*  *EC = electrical conductivity*  *ORs = odds ratios* | |

*Supplementary table 1 – A table showing the general characteristics of the studies included, namely the title, authors, year of publication, study design, country studied, population, exposure(s), comparator(s), outcome(s), as well as the confounders that each study adjusted for and the quality appraisal based on the ROBINS-E [43] Cochrane tool, with a summary of any bias concerns identified during the appraisal process.*

Supplementary figure 2. Dose-response relationship of systolic and diastolic blood pressure in pregnant women with drinking water sodium concentrations.

*Supplementary figure 2 – A graph plotting the values of systolic and diastolic blood pressure (with standard deviations) in pregnant women against drinking water sodium concentration. The two studies selected [11, 13] have comparable populations, exposures and outcomes. With higher salinity of the drinking water, both systolic (SBP) and diastolic blood pressure (DBP) are found to increase linearly, suggesting a (possibly causal) relationship.*

Supplementary figure 3. Dose-response relationship of the odds ratio of developing gestational hypertension and/or preeclampsia in pregnant women with drinking water sodium concentrations.

*Supplementary figure 3 – A graph plotting the odds ratio of developing gestational hypertension and/or preeclampsia (with 95% confidence intervals) in pregnant women against drinking water sodium concentration. The two studies selected [13, 29] have comparable populations, exposures and outcomes. With higher salinity of the drinking water, the odds ratio of these adverse maternal health outcomes increases linearly, suggesting a (possibly causal) relationship.*

Supplementary table 2. Assessment of the risk of bias of individual studies, based on the ROBINS-E Cochrane tool [43].

|  | **Confounding** | **Co-interventions or post-exposure interventions** | **Suitability and quality of exposure measurement and classification** | | **Suitability and quality of outcome measurement and classification** | | **Participants selection process** | **Randomisation process** | | **Missing data** | | **Results reporting** | **OVERALL RISK OF BIAS APPRAISAL** |
| --- | --- | --- | --- | --- | --- | --- | --- | --- | --- | --- | --- | --- | --- |
| *Studies looking at cardiovascular-related health outcomes* | | | | | | | | | | | | | |
| Tuthill et al., 1980 [45] |  |  |  | |  | |  |  |  | |  | | Some concerns |
| Hallenbeck et al., 1981 [46] |  |  |  | |  | |  |  |  | |  | | Some concerns |
| Armstrong et al., 1982 [51] |  |  |  | |  | |  |  |  | |  | | Low risk |
| Faust, 1982 [47] |  |  |  | |  | |  |  |  | |  | | Some concerns |
| Pomrehn et al., 1983 [48] |  |  |  | |  | |  |  |  | |  | | Low risk |
| Welty et al., 1986 [49] |  |  |  | |  | |  |  |  | |  | | Some concerns |
| Pomeranz et al., 2000 [50] |  |  |  | |  | |  |  |  | |  | | Some concerns |
| Talukder et al., 2016 [12] |  |  |  | |  | |  |  |  | |  | | Low risk |
| Scheelbeek et al., 2017 [23] |  |  |  | |  | |  |  |  | |  | | Low risk |
| Talukder et al., 2018 [28] |  |  |  | |  | |  |  |  | |  | | Some concerns |
| Al Nahian et al., 2018 [26] |  |  |  | |  | |  |  |  | |  | | Low risk |
| Chakraborty et al., 2019 [24] |  |  |  | |  | |  |  |  | |  | | Low risk |
| Naser et al., 2019 [25] |  |  |  | |  | |  |  |  | |  | | Low risk |
| *Studies looking at kidney-related and cardiovascular-related health outcomes* | | | | | | | | | | | | | |
| Shuvo et al., 2020 [21] |  |  |  | |  | |  |  |  | |  | | Low risk |
| Naser et al., 2020 [22] |  |  |  | |  | |  |  |  | |  | | Low risk |
| Rosinger et al., 2021 [15] |  |  |  | |  | |  |  |  | |  | | Low risk |
| *Studies looking at pregnancy-related health outcomes* | | | | | | | | | | | | | |
| Khan et al., 2011 [9] |  |  |  | |  | |  |  |  | | |  | Some concerns |
| Khan et al., 2014 [13] |  |  |  | |  | |  |  |  | | |  | Low risk |
| Scheelbeek et al., 2016 [11] |  |  |  | |  | |  |  |  | | |  | Low risk |
| Dasgupta et al., 2016 [44] |  |  |  | |  | |  |  |  | | |  | Some concerns |
| Naser et al., 2020 [27] |  |  |  | |  | |  |  |  | | |  | Some concerns |
| Thompson et al., 2022 [29] |  |  |  | |  | |  |  |  | | |  | Low risk |
|  | | | | | | | | | | | | | |
|  | Low risk | | | Low risk | | Overall risk of bias is low for this study | | | | | | | |
|  | Probably low risk | | | Some concerns | | Overall risk of bias is slightly concerning for this study, but it probably does not threaten the study’s results | | | | | | | |
|  | Probably high risk | | | Some concerns | | Overall risk of bias is concerning for this study, and it could threaten the study’s results | | | | | | | |
|  | High risk | | |  | |  | | | | | | | |
|  | No information given | | |  | |  | | | | | | | |

*Supplementary table 2 – A table detailing the risk of bias assessment for each one of the studies we included individually, using the criteria of the Cochrane tool ROBINS-E [43] (uncontrolled confounding; co-interventions or post-exposure interventions; suitability and quality of the measurement and classification of the exposure(s) and the outcome(s); participants selection process, randomisation process; bias due to missing data; bias in the results reporting). The last column summarises the overall risk of bias appraisal for the study. The studies are arranged into three categories depending on the type of health outcomes they look at: cardiovascular-related, kidney-related and pregnancy-related.*

Supplementary table 3. Assessment of the quality and strength of the bodies of evidence, based on the Navigation Guide [52].

|  | **Cardiovascular-related health outcomes** | **Kidney-related health outcomes** | **Pregnancy-related health outcomes** |
| --- | --- | --- | --- |
| **Risk of bias** | The risk of bias of the entire evidence body was moderately high, with ten studies being at low risk, and six presenting some bias concerns (which could threaten the results in five of them). | The risk of bias of the entire evidence body was low, as all three studies were at low risk of bias. | The risk of bias of the entire evidence body was moderately high, as only three of the six studies were at low risk of bias; three presented some concerns (which could threaten the results in one of them.) |
| **Indirectness** | The outcomes were assessed directly in the concerned populations (i.e. human populations chronically exposed to high-sodium drinking water). | The outcomes were assessed directly in the concerned populations (i.e. human populations chronically exposed to high-sodium drinking water). | The outcomes were assessed directly in the concerned populations (i.e. human populations chronically exposed to high-sodium drinking water). |
| **Inconsistency** | The levels of sodium exposure and the levels used as comparators were extremely variable across studies, and so did the methods to measure and classify the sodium levels. | The levels of sodium exposure and the levels used as comparators were extremely variable across studies, and so did the methods to measure and classify the sodium levels. | The levels of sodium exposure and the levels used as comparators were extremely variable across studies, and so did the methods to measure and classify the sodium levels. |
| **Imprecision** | Confidence intervals and standard deviations were relatively large in several studies, suggesting less precise findings. P-values sometimes were not statistically significant. | Confidence intervals and standard errors were overall narrow and allowed for a confident interpretation of the findings. Most findings were statistically significant. | Confidence intervals and standard deviations were overall narrow and allowed for a confident interpretations of the findings. P-values sometimes were not statistically significant. |
| **Publication bias** | There are no concerns of publication bias, as we found studies of all sizes with positive, negative and null findings. | Only three studies have been published on this health outcome, all of which found water salinity to have an adverse effect on health. It is difficult to comment on publication bias. | There are concerns of publication bias, as there were several smaller studies with positive findings, and two larger studies with negative or null findings. |
| **Large magnitude of effect** | Most of the studies with a positive finding demonstrated a large effect of sodium exposure on adverse cardiovascular outcomes. | Two of the three studies found a large and clear adverse effect of sodium exposure on renal outcomes. One study had more mixed findings. | Most of the studies with a positive finding demonstrated a considerably large effect of sodium exposure on adverse maternal outcomes. |
| **Dose-response** | Five studies demonstrated a dose-response relationship between higher sodium concentrations in drinking water and adverse cardiovascular outcomes; two studies found the reverse dose-response relationship. | One study demonstrated a dose-response relationship; the other two did not focus on this aspect in their analysis. | Only two studies assessed for possible dose-response relationship, and both did find such a relationship. The other studies did not focus on this aspect in their analysis. |
| **Confounding minimises effect** | There is no evidence that any residual confounding would have influenced results in a way to cause a falsely positive result. | There is no evidence that any residual confounding would have influenced results in a way to cause a falsely positive result. | There is no evidence that any residual confounding would have influenced results in a way to cause a falsely positive result. |
| **Overall quality of the body of evidence** | Moderate | Good | Moderate |
| **Overall strength of the body of evidence** | Moderate, due to risk of bias, inconsistency and imprecision. | Inadequate, due to the limited number of studies and inconsistency amongst them. | Moderate, due to risk of bias, inconsistency and risk of publication bias. |
|  |  |  |  |
|  | Suggestive that the body of evidence is of higher quality and/or strength | | |
|  | Suggestive that the body of evidence is probably of higher quality and/or strength | | |
|  | Suggestive that the body of evidence is probably of lower quality and/or strength | | |
|  | Suggestive that the body of evidence is of lower quality and/or strength | | |

*Supplementary table 3 – A table detailing the quality and strength assessment for the bodies of evidence as wholes, using the criteria of the Navigation Guide [52] (risk of bias; indirectness; inconsistency; imprecision; publication bias; large magnitude of effect; dose-response; confounding minimising the effect). The last two lines summarise the overall quality and strength appraisals. The analysis was done separately for each health domain assessed: cardiovascular-related, kidney-related and pregnancy-related outcomes.*

Supplementary table 4. Guideline values of drinking water salinity and limits used for this study.

|  | **Guidelines (recommended values)** | | | | | |
| --- | --- | --- | --- | --- | --- | --- |
|  | **WHO [8]** | **Bangladesh [34, 35]** | | **Jordan [36]** | **US [31, 37]** | **Present study** |
| **Salinity (ppt)** | - | < 0.6 | | - | - | < 0.6 |
| **Sodium concentration (mg/l)** | < 200* | < 200 | | < 400 | < 120** | < 200 |
| **EC (μS/cm)** | - | - | | - | 30-1500 | < 1,000 |
| **TDS (mg/l)** | < 1,000* | < 1,000 | | < 1,500 | - | < 1,000 |
| *EC = electrical conductivity*  *TDS = total dissolved solids*  *US = United States*  *ppt = parts per thousands* | | | *mg/l = milligrams per litre*  *μS/cm = micro-Siemens per centimetre*  ** only for palatability reasons (not for health reasons)*  *** benchmark (non-enforceable)* | | | |

*Supplementary table 4 – As no consensus exists on the highest safe limit of water sodium concentration, we inferred the threshold used in this review by comparing the drinking water guidelines of different institutions and governments.*

Supplementary table 5. Search strategy (template used in Medline).

| **Line** | **Searches** |
| --- | --- |
| 1 | Drinking Water/ |
| 2 | ((drink* or potable or consum* or intake* or use* or usage*) adj2 (water* or groundwater* or ground water* or freshwater* or fresh water*)) |
| 3 | 1 or 2 |
| 4 | Salinity/ or Sodium/ or Sodium Chloride/ or Saline Waters/ |
| 5 | ((salin* or salt* or sodium) adj3 water*) |
| 6 | 4 or 5 |
| 7 | Pre-eclampsia/ or Eclampsia/ or exp Hypertension, Pregnancy-Induced/ or Birth Weight/ or Premature Birth/ or exp Infant, Premature/ or exp Infant, Low Birth Weight/ or Maternal Death/ or Perinatal Death/ or Pregnancy Outcome/ or Stillbirth/ or Pregnancy Complications/ or Abortion, Spontaneous/ or exp Fetal Death/ or exp pregnancy complications, cardiovascular/ |
| 8 | (((complication* or hypertens* or outcome* or cardiovascular) adj2 pregnan*) or (abortion* adj1 spontaneous) or miscarriage* or stillbirth* or still birth* or ((fetal or maternal or perinatal or neonatal) adj (mortalit* or death*)) or prematurity or preterm or pre term or (prematur* adj3 (infant* or baby or babies or birth* or born or deliver*)) or (low adj3 (birth weight* or birthweight*)) or lbw or vlbw or elbw or pre?eclampsia or eclampsia or (gestation* adj2 hypertens*)) |
| 9 | 7 or 8 |
| 10 | Hypertension/ or Blood Pressure/ or Arterial Pressure/ or Cardiovascular Diseases/ or Prehypertension/ or Heart Diseases/ or Coronary Disease/ or Arrhythmias, Cardiac/ or Ventricular Fibrillation/ or Atrial Fibrillation/ or Heart Arrest/ or Myocardial Infarction/ or Myocardial Ischemia/ or Ischemic Attack, Transient/ or Heart Failure/ or Acute Coronary Syndrome/ or Angina Pectoris/ or Vascular Diseases/ or Aneurysm/ or Aortic Aneurysm/ or "Embolism and Thrombosis"/ or Embolism/ or Thromboembolism/ or Thrombosis/ or Peripheral Vascular Diseases/ or Peripheral Arterial Disease/ or Arterial Occlusive Diseases/ or Arteriosclerosis/ or Arteriosclerosis Obliterans/ or Atherosclerosis/ or Chronic Limb-Threatening Ischemia/ or Intermittent Claudication/ or Death, Sudden, Cardiac/ or Cerebrovascular Disorders/ or Brain Ischemia/ or Brain Infarction/ or Dementia, Vascular/ or Intracranial Arterial Diseases/ or "Intracranial Embolism and Thrombosis"/ or Stroke/ or Ischemic Stroke/ or Embolic Stroke/ or Thrombotic Stroke/ |
| 11 | (hypertens* or ((blood or arterial) adj pressure*) or bloodpressure or prehypertens* or ((heart or cardi* or coronary or vascular or arterial) adj disease*) or cardiovascular accident* or CVA or ((cardiac or heart) adj arrythm*) or ((ventricular or atrial) adj fibrillat*) or ((heart or cardi*) adj (arrest* or failure* or death*)) or asystole* or ((myocardial or heart or brain or cerebrovascular) adj (infarct* or isch?emi* or thrombo* or emboli* or apoplexy)) or ((transient isch?emic or heart or brain) adj attack*) or coronary syndrome* or angina* or angor pectoris or aneurysm* or emboli* or thrombo* or arteriosclero* or atherosclero* or ((arter* or vascular) adj (occlu* or obstruct*)) or ((leg or limb) adj (isch?emi* or occlu*)) or intermittent claudicat* or cerebrovascular disorder* or vascular dementia* or stroke*) |
| 12 | 10 or 11 |
| 13 | kidney diseases/ or hypertension, renal/ or renal insufficiency/ or renal insufficiency, chronic/ or nephritis/ or glomerulonephritis/ or "chronic kidney diseases of uncertain etiology"/ or kidney failure, chronic/ or proteinuria/ or albuminuria/ |
| 14 | (((kidney or renal) adj (disease* or failure* or insufficienc*)) or renal hypertension or CKF or CKD or CRF or CRD or ESRF or ESKF or ESRD or ESKD or nephropath* or nephrit* or glomerulo* or glomerular disease* or (chronic adj (kidney or renal)) or proteinuria* or albuminuria* or m?croalbuminuria*) |
| 15 | 13 or 14 |
| 16 | 9 or 12 or 15 |
| 17 | 3 and 6 and 16 |
| 18 | exp animals/ not humans.sh. |
| 19 | 17 not 18 |

*Supplementary table 5 – A table showing the template of the search strategy used. The model shown here was the one used in Medline, and was adapted with a librarian’s guidance to fit the other databases searched (Embase, Scopus, Web of Science and Global Health).*

Supplementary table 6. Inclusion and exclusion criteria.

|  | **Inclusion criteria** | | **Exclusion criteria** | |
| --- | --- | --- | --- | --- |
| **Population** | - Humans, including: - adults; - elderly people; - pregnant women; - adolescents and young people; - children; - neonates and infants. | | - Non-human animals | |
| **Exposure** | - Chronic consumption of high-salinity water, defined as either: - salinity > 0.6 ppt; - sodium concentration > 200 mg/l; - EC > 1000 μS/cm; - TDS > 1000 mg/l. | | - No measurement of the drinking water salinity, sodium concentration, EC or TDS (measurement of chloride alone was not accepted) - Short-term consumption of high-salinity water (for example, assignment to a ‘high salinity group’ for the duration of the study) | |
| **Comparator** | - Chronic consumption of water with levels of salinity, sodium, EC or TDS falling either: - below the values defined as ‘high salinity’; - in the ‘high-salinity’ category but at lower levels than the exposed group. - People not reporting the assessed health outcome in the exposed group | | - No control group based on either: - water salinity levels; - health outcome. | |
| **Outcome** | - Assessment of health outcomes related to cardiovascular health, such as: - blood pressure or (pre)hypertension; - heart disease or failure; - atrial or ventricular arrhythmias or fibrillation; - myocardial ischaemia or infarction, transient ischaemic attack or angina; - brain ischaemia or infarction, stroke, or vascular dementia; - (aortic) aneurysm; - (peripheral) vascular or arterial disease, embolism, thrombosis, arteriosclerosis, or limb ischaemia. - Assessment of health outcomes related to renal function, such as: - kidney disease, insufficiency or failure; - renal hypertension; - (glomerulo)nephritis, proteinuria or albuminuria. - Assessment of health outcomes related to pregnancy, such as: - gestational hypertension, (pre)eclampsia or cardiovascular complications of pregnancy; - premature birth or low birthweight; - maternal, perinatal or infant death; - stillbirth or miscarriage. | | - No assessment of a health outcome related to either: - cardiovascular health; - renal function; - pregnancy (either maternal or infant health). | |
| **Study design** | - Case-control studies - Cohort studies - Cross-sectional studies - Ecological studies - Observational studies - Descriptive studies | | - Case reports - Experimental studies, including randomised controlled trials - Reviews, including scoping reviews, literature reviews, systematic reviews and meta-analyses - Qualitative studies - Commentaries - Conference abstracts - Grey literature | |
| **Other** | - Published in English or English translation available | | - Not published in English or no translation available | |
| *EC = electrical conductivity*  *TDS = total dissolved solids* | | *ppt = parts per thousands*  *mg/l = milligrams per litre* | | *μS/cm = micro-Siemens per centimetre* |

*Supplementary table 6 – A table displaying the inclusion and exclusion criteria for the screening process of the review, as per the PECOS criteria.*
